# Fabrication and appraisal of axitinib loaded PEGylated spanlastics against MCF- 7 and OV- 2774 cell lines using molecular docking methods and in-vitro study

**DOI:** 10.1371/journal.pone.0325055

**Published:** 2025-07-01

**Authors:** Randa Mohammed Zaki, Basmah Nasser Aldosari, Layla A. Alkharashi, Alyaa Alsalhi, Obaid Afzal, Bodoor Ghanem Alanazi, Maha Alsunbul, Rawan Bafail, Fatma I. Abo El-Ela, Hanan O. Farouk

**Affiliations:** 1 Department of Pharmaceutics, College of Pharmacy, Prince Sattam Bin Abdulaziz University, Al-Kharj, Saudi Arabia; 2 Department of Pharmaceutics and Industrial Pharmacy, Faculty of Pharmacy, Beni-Suef University, Beni-Suef, Egypt; 3 Department of Pharmaceutics, College of Pharmacy, King Saud University, Riyadh, Saudi Arabia; 4 Department of Pharmacology and Toxicology, College of Pharmacy, King Saud University, Riyadh, Saudi Arabia; 5 Department of Pharmaceutical Chemistry, College of Pharmacy, Prince Sattam Bin Abdulaziz University, Al-Kharj, Saudi Arabia; 6 Department of Pharmaceutical Sciences, College of Pharmacy, Princess Nourah Bint Abdulrahman University, Riyadh, Saudi Arabia; 7 Department of Pharmaceutics and Pharmaceutical Industries, College of Pharmacy, Taibah University, Al-Madinah Al-munawwarah, Saudi Arabia; 8 Department of Pharmacology, Faculty of Veterinary Medicine, Beni-Suef University, Beni-Suef, Egypt; 9 Department of Pharmaceutics, Faculty of Pharmacy, Nahda University, Beni-Suef, Egypt; University of Michigan, EGYPT

## Abstract

Axitinib is a second-generation tyrosine kinase inhibitor that works by selectively inhibiting vascular endothelial growth factor receptors (VEGFR-1, VEGFR-2, VEGFR-3). Through this mechanism of action, axitinib blocks angiogenesis, tumor growth and metastases and therefor it shows significant promise as a chemotherapeutic agent for various types of cancer. Nevertheless, the clinical efficacy of this substance is hindered by its restricted solubility in water and inadequate stability. To address these challenges, we developed spanlastics with polyethylene glycol (PEG) to improve the efficacy and stability of axitinib against breast and ovarian tumor malignancies in a targeted manner. Moreover, the study conducts a thorough examination of the interactions between the ligand Axitinib alone or after coating with PEG and a diverse array of protein types in breast (Dopamine, VEGFR) and ovarian cancer (EGFR, BCL-xL). The fabrication of axitinib- spanlastics was achieved through a thin-film hydration method. The evaluation of the impact of formulation factors on the features of nanovesicles was conducted using the I- optimal design. Subsequently, the optimum formulation was calculated. The optimal formulation was coated with polyethylene glycol (axitinib-PEG-spanlastics). An *in vitro* assessment was computed to evaluate the efficiency of the optimized axitinib-PEG-spanlastics against the MCF-7 breast cancer cell line and the OV-2774 ovarian cancer cell line. The optimized axitinib-PEG-spanlastics formulation exhibited a diameter of 563.42 ± 8.63 nm, accompanied by a zeta potential of −46.44 ± 0.09 mV. The formulation demonstrated an 84.32 ± 3.64% entrapment percent and a cumulative release of 73.58 ± 3.37% during a 4-hour period. The results obtained from the WST-1 assay showed a significant decrease in the percentage of cell survival, reaching 50% at a concentration of 0.68 µM for the PEG-spanlastics. In contrast, the axitinib free drug suspension exhibited 50% cell survival at a concentration of 1.1 µM in the breast cancer (MCF-7) cell line. In MCF-7 cells, the percentage of apoptotic cells generated by axitinib-PEG-spanlastics compared to the free drug suspension was 70.76 ± 4.971% vs. 32.6 ± 1.803%, while in OV-2774 cells, it was 43.55 ± 4.243% vs. 24.44 ± 4.950%. These results propose that Axitinib-PEG-spanlastics have the potential to be a successful nanoplatform for targeting breast and ovarian cancer and effectively managing tumors.

## 1. Introduction

The global prevalence of cancer is experiencing a concerning upward trend, resulting in an annual mortality rate above 8.2 million individuals [1]. Malignant neoplasms pose significant risks to human health, and the available options for managing them are severely limited [2]. The primary factor contributing to the rising mortality rates caused by cancer is the inadequate targeting of tumors and the significant adverse effects linked to the majority of anti-cancer medications.

Numerous chemotherapeutic agents have historically been employed in the treatment of ovarian and breast cancers, despite the increasing apprehensions surrounding their utilization. These agents include antimetabolites (methotrexate and 5 fluorouracil), taxanes (docetaxel and paclitaxel), platinum-based drugs (cisplatin and oxaliplatin), and alkylating agents (cyclophosphamide) [2]. The concerns encompass a range of issues, such as the occurrence of severe systemic side effects, the accumulation of toxicity in different biological critical organs, the induction of cell death in normal cells, inadequate targeting, and low bioavailability profiles [2]. Significantly, the utilization of chemotherapeutic agents in the management of cancer is consistently associated with an elevated occurrence of tumor relapse and recurrence, as well as the development of multiple drug resistance [2,3].

Axitinib, a receptor tyrosine kinase inhibitor of the second generation, operates by stabilizing the receptor kinase domain in an inactive conformation, thereby inhibiting VEGF receptors VEGFR-1, VEGFR-2, and VEGFR-3 at picomolar dose [4]. Axitinib has been shown to have potential efficacy in treating neovascularization in corneal, retinal, and choroidal tissues in various animal models, including mice, rats, and rabbits [5–10]. The study conducted by Nakano et al. and Kansara et al. has shown evidence of the biological efficacy of suprachoroidally administered axitinib in a laser-induced choroidal neovascularization model, as well as in a retinal vascular leakage model, using rats and pigs [10,11].

Due to its strong ability to block all types of VEGFR, axitinib may offer a significant advantage over existing anti-VEGF-A drugs. This can result in the increased expression of VEGF-C and VEGF-D [12,13]. The increased expression of additional VEGF ligands may contribute to tachyphylaxis, a type of treatment resistance, and could result in clinically refractory patients. Studies have shown that axitinib is more efficient than a VEGF-A inhibitor in inhibiting angiogenic sprouts, suggesting that pan-VEGF inhibition may have a potential advantage over concentrated VEGF-A inhibition [6]. In a recent phase II clinical research conducted by Parravano et al., it was observed that broad VEGF inhibition resulted in a statistically significant improvement in visual outcomes in individuals with neuroatopic musculoskeletal disorder (nAMD) compared to focused VEGF-A inhibition [14].

Despite such remarkable pharmacological traits, axitinib in vivo exploitation is overtly hampered by its sparse aqueous solubility, rapid elimination from the site of action, limited physiological milieu stability, poor permeability, extensive first-pass metabolism, necessitating frequent dosing and compromising patient adherence. Notably, axitinib oral bioavailability (33%) [9,15]. This has sparked significant interest in the advancement of diverse and inventive medication delivery technologies to address these constraints.

The efficiency of axitinib against many cancer types was boosted through its encapsulation into different types of nanoparticles, including mesoporous silica and nanohybrid liposomal-based nanoparticles [16,17]. Nevertheless, the existing body of literature reveals a scarcity of studies investigating the utilization of axitinib-loaded nanoparticles as a therapeutic approach for breast and ovarian cancer. This research gap holds significant potential for further exploration and serves as the foundation for the present investigation [18–23].

Niosomes are drug carriers that exhibit colloidal structure and are created through the self-assembly of non-ionic surfactants. Their chemical stability and capacity to improve medication transport to different body regions have garnered considerable interest [24]. Spanlastics, also known as elastic niosomes, are a recent addition to drug delivery systems that utilize vesicles. Non-ionic surfactant vesicles, such as niosomes, bear resemblance to these entities; nevertheless, they diverge in their inclusion of an edge activator. The ocular drug delivery systems were initially documented by Kakkar and Kaur in 2001. However, subsequent research has revealed their use in delivering drugs to different anatomical regions [25]. An edge activator incorporated within the spanlastics bilayers mitigates the adverse effects of the medications by specifically targeting the tumor through an increased permeability and retention (EPR) effect. Nevertheless, the achievement of a high level of anti-tumor efficacy and the elucidation of the tumor targeting mechanism associated with this strategy continue to pose a substantial obstacle. In the current study, our objective was to encapsulate axitinib into nanoparticles, denoted as axitinib- Np, using Polyethylene glycol (PEG) as the encapsulating agent. The water-soluble polyethylene glycol (PEG) is a polymer that is FDA-approved, non-toxic, non-immunogenic, and readily available in the market. Polyethylene glycol (PEG), conversely, is an amphiphilic polymer that exhibits remarkable biocompatibility and is commonly employed in biomedical contexts. In addition, the process of conjugation with polyethylene glycol (PEG) serves to limit the traversal of the blood-brain barrier, hence mitigating the neurotoxic effects commonly associated with unbound medications or phytochemicals. Moreover, this conjugation technique also extends the duration of circulation for these pharmaceuticals or phytochemicals [26]. Camptothecin, doxorubicin, and paclitaxel have been conjugated to PEGs, and these conjugates, particularly camptothecin, are currently undergoing clinical studies [27–29]. Due to the potential efficacy of these features in the clinical use of the medicine, we opted to utilize PEG instead of other polymers for the nano-encapsulation of axitinib.

Notwithstanding the aforementioned captivating characteristics of PEG-spanlastics, their capacity to augment the effectiveness of anticancer medications and address their limitations remains untapped. Furthermore, a meticulous examination of the literature reveals a lack of published studies investigating the utilization of PEG-spanlastics for the treatment of breast and ovarian cancer. This study aimed to develop targeted PEG-spanlastics nano vesicular systems for axitinib delivery in order to address its unfavorable physicochemical characteristics and improve its effectiveness in combating breast and ovarian cancer cell lines. The cytotoxic efficacy of axitinib-loaded PEG-spanlastics was assessed *in vitro* against the breast cancer cell line MCF-7 and the ovarian cancer cell line OV-2774 using various methodologies.

## 2. Materials and methods

### 2.1. Materials

Axitinib, Span 60 (sorbitan monostearate), Tween 80 (polyoxyethylene sorbitan monooleate) and sodium deoxycholate (SDC) were acquired from Sigma-Aldrich (St. Louis, MO). PEG-6-stearate (Superpolystate®) was donated by Gattefossè (Lyon, France). Additional analytical-grade compounds and solvents were procured from El-Nasr Pharmaceutical Company (Cairo, Egypt).

### 2.2. Design and optimization of the experiments

For the purpose of statistical optimization of axitinib-loaded spanlastic formulation, I optimum design was implemented (Table 1) using design expert software (Version 12, Stat-Ease Inc. Minneapolis, MN, USA). Span 60 amount ranged from 300 to 400 mg, edge activator amount ranged from 100 to 200 mg, and edge activator type varies between tween 80 or SDC, were the independent variables as shown in Table 1. Entrapment efficiency (EE)(Y1), vesicle size (Y2), and zeta potential (Y3) were dependent variables. In all formulations, the AXT concentration was kept constant at 0.1 (%w/v). In order to achieve the formulation with the highest desirability, the optimization process was conducted by considering a minimum value of VS and a maximum absolute value of ZP, as well as EE% [30]. The independent variable levels and experimental runs generated by the I optimum design are presented in Table 2.

**Table 1:** I Optimal design for optimization of Axitinib loaded Spanlastics.

Independent variables	Levels	
	Low	High
Span 60 amount (X1)	300	400
Edge activator amount (X2)	100	200
Type of edge activator (X3)	Tween 80	SDC
**Dependent values (Responses)**	**Desirability**	
EE% (Y1)	Maximize	
Vesicles size (Y2)	Minimize	
Zeta potential (Y3)	Maximize	

**Table 2:** Composition of different formulations with their responses in I optimal design for optimization of Axitinib loaded Spanlastics.

Formula	Independent Variables			Dependent Variables			
	Span 60 amount (mg) (X1)	Edge activator amount (mg) (X2)	type of edge activator (X3)	EE% (Y1)	Vesicle size (nm) (Y2)	Zeta potential (mv) (Y3)	PDI
SLs 1	350	150	SDC	81.3 ± 1.34	626.6 ± 5.23	−37.2 ± 0.234	0.374 ± 0.013
SLs 2	350	150	Tween 80	73.8 ± 2.04	478.4 ± 8.86	−22.2 ± 0.321	0.532 ± 0.021
SLs 3	400	200	Tween 80	87.4 ± 2.13	713.2 ± 7.98	−25.9 ± 0.410	0.482 ± 0.020
SLs 4	350	150	SDC	79.8 ± 1.05	588.5 ± 7.67	−43.1 ± 0.763	0.473 ± 0.014
SLs 5	300	200	Tween 80	83.2 ± 1.65	643 ± 6.83	−23.3 ± 0.372	0.485 ± 0.112
SLs 6	350	150	SDC	81.9 ± 2.89	632.5 ± 8.97	−38.5 ± 0.654	0.561 ± 0.018
SLs 7	300	200	SDC	89.2 ± 2.64	765.0 ± 5.89	−41.9 ± 0.562	0.382 ± 0.036
SLs 8	400	100	Tween 80	71.1 ± 1.72	387.8 ± 5.12	−25.7 ± 0.372	0.352 ± 0.043
SLs 9	400	100	SDC	73.3 ± 2.65	474.8 ± 4.99	−45 ± 0.326	0.364 ± 0.025
SLs 10	400	200	SDC	83.9 ± 1.03	653.5 ± 3.75	−41.7 ± 0.639	0.274 ± 0.018
SLs 11	350	200	Tween 80	84.3 ± 1.08	662.6 ± 4.02	−26 ± 0.482	0.280 ± 0.045
SLs 12	350	150	SDC	80.2 ± 1.86	606.8 ± 7.23	−38.5 ± 0.372	0.471 ± 0.034
SLs 13	400	100	SDC	75.2 ± 1.11	535.4 ± 6.01	−42.5 ± 0.498	0.372 ± 0.022
SLs 14	300	100	Tween 80	65.5 ± 1.35	223.2 ± 3.45	−23.1 ± 0.584	0.231 ± 0.052
SLs 15	300	100	SDC	77.2 ± 2.65	583.5 ± 8.99	−34.1 ± 0.764	0.553 ± 0.027
SLs 16	400	150	Tween 80	85.7 ± 2.77	689.1 ± 8.32	−31.6 ± 0.642	0.512 ± 0.016
SLs 17	400	200	SDC	90.3 ± 2.89	784.4 ± 9.56	−43 ± 0.831	0.587 ± 0.021
SLs 18	350	100	Tween 80	67.7 ± 1.03	299.3 ± 4.01	−18.1 ± 0.253	0.254 ± 0.044
SLs 19	300	150	Tween 80	75.8 ± 1.22	539.2 ± 5.88	−27.3 ± 0.164	0.377 ± 0.031
Optimized formula	400	119.151	SDC				

### 2.3. Preparation of axitinib- loaded spanlastics

The fabrication of spanlastics was conducted using the previously described thin film hydration process, with minor modification [31]. 0.01 gm of Axitinib and the specified amounts of span 60 and edge activator (Tween 80 or SDC) (Table 2) were dissolved in 10 ml chloroform, a rotary evaporator (Heidolph Laborota 4000 Series, Heizbad, Germany) at 90 rpm evaporated the organic solvent at 60 °C under vacuum, forming a thin film on the flask wall. To hydrate the film, 10 ml of pH 7.4 phosphate-buffered saline (PBS) was added to the flask. The flask was then rotated in a water bath at 60°C for 30 minutes under normal pressure. The spanlastics suspension was then sonicated for 10 min to reduce the particle size and finally maintained at 4 °C overnight to anneal the produced bilayers. The resulting compositions were stored in the refrigerator for additional examination.

### 2.4. Characterization of axitinib- loaded spanlastics

#### 2.4.1. Determination of drug entrapment efficiency percent (EE%).

The indirect method was employed to determine the percentage of drug entrapped within spanlastics. The unentrapped amount of drug was deducted from the total beginning amount of the drug utilized to make the spanlastics [32]. The unentrapped drug was separated from the produced spanlastics by centrifugation at 14000 rpm for 2 hours at 4 °C using a cooling centrifuge (Sigma Laborzentrifugen, Osterode am Harz, Germany). To achieve full removal of the un-entrapped drug, the separated spanlastics were washed twice in two distinct processes by re-suspending the recovered vesicles in PBS pH 7.4. The spanlastics obtained were subjected to another round of centrifugation. The supernatants were periodically separated and subjected to spectrophotometric analysis at a wavelength of 259 nm (Spectronic Genesys® with Winspec Software, Spectronic, USA). This analysis aimed to quantify the quantity of axitinib present after appropriate dilution, employing a standard calibration curve. The calculation of entrapment efficiency (%) was performed using the equation [32]:


$$EE\;\%=\frac{Total\;\;amount\;of\;drug\;added-unentrapped\;\;drug\;amount}{Total\;\;amount\;of\;drug\;added}X100$$ (1)

#### 2.4.2. Determination of vesicle size, polydispersity index and zeta potential.

The size and size distribution of spanlastics were measured using the Zetasizer Nano 7.11 (Malvern Instruments, Malvern, UK). In summary, the spanlastics preparations were diluted at a ratio of 1:50 using distilled water and subsequently subjected to analysis at a temperature at 25 °C [33]. For each preparation, three replicates were taken for the measurements, and the average values were then computed. The polydispersity index (PDI) was determined using the same instruments and was utilized as a metric for assessing the distribution of particle sizes [34].

### 2.5. Formulation of axitinib-PEG-spanlastics

Based on the optimization process by Design Expert software, the optimal formulation was selected and further prepared with PEG. The axitinib-PEG-spanlastics were prepared by incorporating PEG (5% w/v) into PBS, using the same manner a described section 2.3.

### 2.6. Characterization of axitinib-PEG-spanlastics

#### 2.6.1. VS, ZP, and EE% analysis.

As previously reported, the VS, ZP, and EE% of axitinib-PEG-spanlastics were assessed.

#### 2.6.2. Differential scanning calorimetry (DSC) analysis.

An investigation was conducted using DSC (DSC50 Shimadzu, Kyoto, Japan) to examine the thermal characteristics of pure axitinib, its components, and the optimal axitinib-PEG-spanlastics. Crimping was performed on five mg-samples in a standard aluminum pan heated at a constant rate of 5 °C/min from 25 to 300 °C while being purged of argon at a constant rate of 20 ml/min [35].

#### 2.6.3. X-Ray diffraction (XRD).

The samples’ powder x-ray diffraction was analyzed using an Ultima IV diffractometer (Rigaku, College of Pharmacy – King Saud University, KSA) with a scan speed of 1.00 degrees per minute and a range of 3 − 140° 2θ. The anode of the tube was Cu, monochromatized with a graphite crystal, with a Ka value of 0.1540562 nm. At 40 kV of tube voltage and 40 mA of tube current, the pattern was acquired in step scan mode (0.02° step size, 1 second counting time per step) [36].

#### 2.6.4. Morphological evaluation.

Axitinib-PEG-spanlastics were evaluated for their morphology using a transmission electron microscope (JEM1400, Jeol, Tokyo, Japan). A single droplet of the optimum formula, diluted at a ratio of 1:10, was applied onto a carbon-coated copper grid. The droplet was then let to dry and subsequently stained with a 2% phosphotungestic acid aqueous solution as a negative stain. Ultimately, it is scrutinized utilizing a transmission electron microscope (TEM) operating at 80 kV [37].

#### 2.6.5. *In vitro* drug release.

The dialysis bag diffusion technique, with a molecular weight cut-off of 10,000 Da, was used to conduct the in vitro drug release investigation of axitinib suspension and axitinib-PEG-spanlastics. A 20 mL release medium containing 0.1% (v/v) Tween 80 was added to the dialysis tube that contains 1 ml of the formula. The release medium was made of PBS with a pH of 7.4 [38]. The contents were maintained at 37°C and swirled continuously at 150 rpm using a magnetic stirrer. One milliliter of sample was taken and replaced with new release medium to achieve the sink conditions at 1, 2, 3, and 4-hour intervals according to the planned schedule. Using UV spectrophotometry at a wavelength of 259 nm, the concentration of the drug was measured following appropriate dilution of the samples. The results are presented as mean values ± SD. The data were fitted into various kinetic models: zero-order, first-order, second-order, and Higuchi models. The correlation coefficient (R2) was determined for each case.

#### 2.6.6. Stability studies.

A stability investigation was conducted on the formulation of axitinib-PEG-spanlastics by storing it in a glass vial at a temperature of 4 °C. Samples were collected from the glass vial at time intervals of 0 and 1 month, and subsequently subjected to analysis for mean VS, EE%, and ZP. Furthermore, the physical characteristics were assessed for the presence of aggregation, separation, or precipitation.

### 2.7. Cytotoxic assay against t breast cancer cell line MCF-7 and ovarian cancer cell line OV-2774

#### 2.7.1. Cells and cell culture.

Breast cancer cells (MCF-7) and ovarian cancer cells (OV-2774) were obtained from ATCC, authenticated by ATCC using short tandem repeat profiling, propagated, expanded, and frozen immediately into numerous aliquots after arrival. The revived cells were used within 10–12 passages and for a maximum of three months after their revival. The cells were grown in DMEM/F2 (1:1) from GIBCO®, InvitrogenTM, containing 10% FBS and 1% antibiotics/antimycotic. Detection of mycoplasma contamination has been carried out regularly using Universal Mycoplasma Detection Kits (ATCC 30‐1012K). Sigma-Aldrich provided all supplements except for the antibiotic and antimycotic/antimycotic solutions, which were obtained from Gibco. The cells were maintained at 37°C in a humidified incubator containing 5% CO2.

#### 2.7.2. Cell proliferation assay.

The optimal Axitinib-PEG-spanlastics and Axitinib spanlastics were compared to the free drug suspension using the two cell lines mentioned above using the WST1 assay (Sigma-Aldrich). 5000 cells were seeded into 96-well microtiter plates in a final volume of 100 liters of a suitable culture medium and incubated overnight. 0.4-fold serial dilutions were added to all formulations of axitinib (0.4–1.6 µM) on MCF-7 and10 fold serial dilutions (10–40 μM) were added to OV-2774 for an additional 48 hours. After cell treatment, WST1 reagent (ab155902) was added and incubated for four hours at 37°C. In the control wells, 100 µl of culture medium were combined with 10 µl of WST-1. ELISA microplate readers (Thermo Fisher Scientific, USA) were used to measure the intensity of formazan dye at 440 nm. In order to calculate the cytotoxicity percentage, the following formula was used;


$$\;\%\;Cytotoxicity=\frac{Control-treated\;sample\;x100}{Control}$$ (2)

#### 2.7.3. Apoptosis measurement by flow cytometry.

An Annexin V Apoptosis Detection Kit (Multisciences, Hangzhou, China) was utilized to measure apoptosis of MCF-7 and OV-2774 cells. After treatment with the determined IC50 dose for 72 h, cells were trypsinized and washed with PBS, and resuspended in the binding buffer at a concentration of 106 cells/ml. Subsequently, 5 μl of Annexin V-FITC and 5 μl of propidium iodide (PI) were added and vortexed gently. Cells were then and incubated for 15 minutes at room temperature. Apoptotic rate was analyzed using flow cytometry (NovoCyte, USA) and the data was analyzed using NovoExpress software.

### 2.8. Molecular docking

#### 2.8.1. Different web sites used for Ligand and Protein Receptor preparation.

Axitinib and PEG were tested against the anticipated protein on breast and ovarian cancer cells, as well as their 3D and 2D binding affinity and bonding. The 3D SDF X-ray crystal structure of Axitinib was received from PubChem (https://pubchem.ncbi.nlm.nih.gov/compound/3086069).

Uni-prot (https://www.uniprot.org/uniprotkb/Q12906/entry) and the protein data bank provided a homology model of breast (Dopamine, VEGFR) and ovarian cancer (EGFR, BCL-xL-Receptors). The human gene bank code and ID were verified on https://www.genecards.org/cgi-bin/carddisp.pl?gene. After identifying the active site or pockets of interactions from the CB.DOCK2 site and the Deep site, these existent structures were used as mooring targets. ModRefiner (https://zhanggroup.org/ModRefiner/) increased protein quality by improving 3D model structures. Deep Site Server predicted active sites (https://www.deepsite.ai/) and CB.DOCk2 verified them more accurately (https://cadd.labshare.cn/cb-dock2/php/blinddock.php#job_list_load).

#### 2.8.2. Preparation and computations of ligand.

The PubChem database was used to obtain the ligands, specifically Axitinib and PEG, in 3D-sdf format. The Avogadro 1.2.0 software 25 and an MMFF94 force field were employed to execute the energy-minimization procedure on their 3D structures. The ligand, which is presently stored in pdb format, will be analyzed on Auto Duck Vienna following the protein preparations. The ligand will be prepared for visualization and interactions on the Biovia 2021 program by performing this analysis in pdbqt.

#### 2.8.3. Preparation and computations of protein receptor.

A three-dimensional (3D) model of the diverse receptors was obtained in order to generate proteins. The entire protein’s alpha fold structure, XRD, and all chain types are included in the PDB structure that Uni-prot has downloaded. The unique protein was prepared by removing water molecules and adding hydrogen atoms to the unoccupied valences of the heavy atoms before docking. The protein’s structure was examined to identify any missing atoms that required the addition of KOLLMAN charges. Any sections that were incomplete were rectified. The appropriate binding site (Pockets), which comprises active sites of interaction with the both ligands, was identified by utilizing the Define and Edit Binding Site protocol in conjunction with the deep site and CB-DOCK2 server. The proteins were classified as receptors. The Auto Dock Vina software was employed to conduct the docking simulations, which were conducted on a grid frame of 30 × 30 × 30. The active site was estimated and determined by utilizing a grid box that measured 30 x 30 x 30 along the x, y, and Z axes. The grid box was then centered at the precise center of the active site. The active sites were subsequently identified using the precise amino acids obtained from the CB-Dock2. Dopamine receptor (Y:90, I:212, L:216, R:220, K:221, A:376, V1071, Y:1088 & I:1100). VEGFR (D:34, F:36, P:40, S:50, A:195, V254, and F:288). BCL-xL; F:16, K:20, K:24, D:99, E:102, F:109, I:118, F:150, D:160, M:163), all in the A-chain. EGFR (L:38, E:60, R:80, L:120, F:230, K:260 & H:280). The sites that were identified as active were eliminated once they were confirmed. The grid box’s dimensions were recorded for future reference, and the binding affinity between the ligand and receptor was determined in the configuration file. Ultimately, the protein was encoded in pdbqt format to facilitate docking with the ligand using the visualization DISCPVERY STUDIO (BIOVIA 2021) program.

#### 2.8.4. Protein–ligand interactions.

The server software was employed to visualize and analyze the protein–ligand interactions following the determination of the binding affinity between the ligand and receptor. The ligand was subsequently designated as Axitinib and PEG in pdbqt format. The receptor was designated as a diverse array of proteins and introduced in pdbqt format. The receptor was designated for each protein twice: once with the ligand and again with the standard medication. The ligand and receptor’s interactions were determined by integrating all amino acids, angles, and atom distances in the desired format. The following interactions were examined: H-bonding, hydrophobic interaction, salt bridge between covalent bonds, aromatic ring center, charge center, and π-stacking (parallel and perpendicular). The binding affinity and free energy (ΔG) were calculated to assess the protein–ligand interaction. In order to investigate interactions, all interactions were recorded in both 3D and 2D at varying dimensionalities.

### 2.9. Statistical analysis

The information was reported as the mean ± standard deviation. A p-value less than 0.05 was considered to indicate significance. Quantification of the western blot bands was performed utilizing the Image J computer software (NIH, USA).

## 3. Results and discussion

### 3.1. Experimental design and optimization

The experimental runs with different amounts of Span 60, edge activator amount, and edge activator type are displayed in Table 2, displaying the outcomes of VS, ZP, PDI, and EE. Table 2 displays the PDI values of axitinib -spanlastics, which varied from 0.231 ± 0.052 to 0.587 ± 0.021. Low PDI values indicate a limited range of sizes and a uniform VS pattern, whereas high PDI values indicate a greater degree of heterogeneity [39]. The analysis of PDI values using ANOVA Type III revealed that the majority of independent factors had insignificant impacts. As a result, the optimization stage did not incorporate PDI. The diverse range of data for the dependent variable indicates that changes in the levels of Span 60, edge activator, and edge activator type can have a significant impact on the properties of spanlastics. Table 3 presents the mathematical equations with coded values that most accurately represent the interplay between the cause and response factors. Design Expert conducted a calculation to determine the level of precision required for assessing the reliability of models employed in navigating the design space [40]. The statistical analysis of response variables demonstrated a level of precision exceeding 4, and the predicted R^2^ values closely aligned with the adjusted R^2^ findings (the difference was less than 0.2), indicating that the model was valid [41,42].

**Table 3:** ANOVA for I optimal design of Axitinib loaded Spanlastics.

Source	EE%		Vesicles size		Zeta potential	
	F value	p value	F value	p value	F value	p value
Model	21.00	< 0.0001	16.91	< 0.0001	24.52	< 0.0001
Span 60 amount (X1) = A	2.14	0.1692	1.15	0.3039	7.21	0.0229
Edge activator amount (X2) = B	97.33	< 0.0001	69.11	< 0.0001	3.03	0.1123
Type of edge activator (X3) = C	13.02	0.0036	17.54	0.0013	157.53	< 0.0001
**Regression equations of different responses**						
EE%	+79.31 + 1.09A + 7.36B + 2.14C-0.0286AB-2.19 AC-1.07BC					
Vesicle size	+572.78 + 18.61A + 144.06B + 57.69C-5.49AB-45.50 AC-40.69BC					
Zeta potential	−31.76-2.07A-1.33B-7.66C + 1.31AB-0.4817 AC + 0.0560BC-4.22 A^2^ + 3.18B^2^					
**Dependent variables**	R2	Adjusted R^2^		Predicted R^2^		Adequate precision
Y1: EE%	0.9131	0.8696		0.7906		15.2075
Y2: Vesicles size (nm)	0.8942	0.8413		0.7114		14.1194
Y3: Zeta potential (mV)	0.9515	0.9127		0.7584		13.1630

#### 3.1.1. Analysis of entrapment efficiency (EE%).

The data presented in Table 2 indicates that the axitinib-spanlastics formulations that were created exhibited a range of EE% values, varying from 65.5 ± 1.35% to 90.3 ± 2.89. The trapping of axitinib within axitinib-spanlastics was significantly impacted by the amount (X2) and type (X3) of the edge activator (p < 0.05) (Table 3). Based on the findings of the regression analysis in Table 3, it was seen that the three independent variables exhibited a positive influence on the EE% outcomes. As shown in S1A and S2A Figs, the entrapment values increased when the levels of Span 60 were raised. This observation can be attributed to the length of the alkyl chain, which facilitates enhanced drug encapsulation [43]. Furthermore, augmenting the amount of Span 60, a substance that forms vesicles, could result in the formation of a more lipophilic milieu to accommodate a greater amount of the hydrophobic medication (axitinib), hence enhancing the encapsulation efficiency (EE%) [44].

Similarly, spanlastic formulations based on SDC showed a significantly greater EE% compared to those containing tween 80, with a p-value < 0.05. This is attributed to its stronger amphiphilic character and detergent properties, which promote the incorporation of hydrophobic drugs into the lipid bilayer or micellar structures. Moreover, the detergent-like properties of SDC help to solubilize and trap hydrophobic drugs within the bilayer more effectively. However, its use needs to be optimized to avoid drug leakage or instability, which can sometimes occur due to its high surface-active nature. According to Karuppusamy and Venkatesan, the EE% values of spanlastics containing SDC showed a significantly greater EE% compared to those containing tween 80 [45]. Moreover, a sodium deoxycholate enhance the encapsulation of hydrophobic drugs into lipid-based delivery systems, resulting in high entrapment efficiency.

The increase in the amount of edge activator had a beneficial effect on the axitinib EE%. This can be attributed to the ability of the edge activator to provide more room for containing additional medication, as well as the formation of a monomolecular edge activator layer to stabilize the interface of the vesicles [46]. The results revealed in this study align closely with the findings reported by Al-Mahallawi et al. (2017), who asserted that a concentration of 20% w/w EA was the most effective for entrapping the medication within spanlastic material [31].

#### 3.1.2. Analysis of vesicle size (VS).

The VS values of the nano vesicular formulations created ranged from 223.2 ± 3.45 to 784.4 ± 9.56 nm, as presented in Table 2. The influence of span 60 and edge activator amount and type on the VS of axitinib-spanlastic formulations is shown in S1B and S2B Figs. Based on the findings of the regression analysis in Table 3, it was seen that the three independent variables exhibited a positive influence on the VS outcomes.

As illustrated in S1B and S2B Figs, VS was increased while increasing the levels of Span 60. The experimental findings indicated that augmenting the surfactant (span 60) amount led to a marginal to modest augmentation in the size of the vesicles. The observed increase in vesicle size is consistent with previous findings and may be attributed to the decrease in the hydrophilic component of the surfactant when low HLB surfactant is present at high concentrations [47]. The results obtained were comparable to those described Albash et al. [48].

With respect to the EA type, the dimensions of spanlastics adhered to the SDC ˃ tween 80 order. The order of the edge activators utilized can be ascribed to the HLB values. SDC has the greatest HLB value of 16.7, while tween 80 has an HLB value of 15 [49]. As previously stated, there is a direct correlation between the HLB value and the size of the spanlastics. Surfactants with greater HLB values have bigger particle sizes, which may be linked to their enhanced surface energy and water absorption [50]. These observations are corroborated by prior investigations [51,52].

Notably, the impact of EA levels on the average VS was found to be significant as shown in Table 3. This can be attributed to the steroid-like cyclic bulky structure and spatial conformation of EA in SDC, which may lead to an increase in vesicle size when incorporated into the bilayer structure [31,53]. Comparable findings were observed by Alaaeldin et al. during the production of thymoquinone-loaded nano vesicular systems [25]. The enlargement of spanlastics as the quantity of tween 80 increases can be ascribed to the repulsive interaction between the surfactant and phospholipid bilayers, as suggested by Stuart et al. and Bnyan et al. [54,55]. The presence of numerous ethylene oxide side chains in the tween 80 structure contributes to increased steric repulsion in the continuous aqueous phase [56]. Similar results were observed by Eleraky et al. when they synthesized nano-vesicular systems laden with curcumin [57].

#### 3.1.3. Analysis of zeta potential (ZP).

The charge of vesicles, denoted as ZP, serves as an indirect metric for assessing the stability of colloidal dispersions [48]. According to Eid et al. (2021), the presence of high surface charge values can potentially hinder the aggregation of nanoparticles by introducing stronger repulsion forces onto their surfaces [58]. Table 2 displays ZP values ranging from −18.1 ± 0.235 to −45 ± 0.326 mV. To prevent any misinterpretation, the variance in ZP will be analyzed based on its absolute value, as all the preparations in this investigation had negative ZP values.

As shown in S1C and S2C Figs, when the level of Span 60 was increased from 300 to 400 mg, ZP rose from (−27.3) to (−31.6) mV. This phenomenon may arise due to the increasing concentration of OH ions, which is employed to attain a high ZP [59]. Furthermore, it has been asserted by Kim et al. that the HLB value of the surfactant has an influence on the competitive adsorption of OH ions at the interface present in the hydration milieu [60]. A decrease in the HLB value of the surfactant (indicating a higher nonpolar interface) leads to an increase in the adsorbed OH, resulting in an elevation in ZP.

The zeta potential values exhibited a progressive increase when the concentration of the edge activator was increased. Such observations is supported by previous research finding [51].

The impact of the edge activator type on the ZP of the prepared spanlastics was determined to be statistically significant (p < 0.05) as shown in Table 3. In relation to SDC surfactants, it was shown that SDC exhibited vesicle formation with notably elevated ZP values in comparison to tween 80. The observed phenomenon can perhaps be attributed to the anionic properties of SDC, which may result in more protection of the (-ve) charge by occupying the surface of the vesicular bilayer and thereby increasing its charge. This stands in contrast to the nonionic nature of tween 80, as noted by Alharbi et al. [51]. According to Ibrahim et al., the presence of (CH2-CH2-O)_n_ in Tweens resulted in the formation of hydrogen bonds with water particles, which subsequently led to a decrease in ZP values [61]. The results of our study are also align with the findings reported by Albash et al. [48].

### 3.2. Formulation optimization

The Design Expert® software provided several proposals that effectively met the required criteria, including the minimum value of VS, maximum value of ZP, and maximum value of EE%. In S3 Fig, the desirability value of the optimized formulation (X1: Span 60: 400 mg, X2: edge activator amount: 119.151 mg (w/v), and X3: edge activator type: SDC) was determined to be 0.731. Table 4 presents the experimental, anticipated, and predicted errors pertaining to the response variables of the best axitinib-spanlastics dispersion. Furthermore, the calculated prediction error percentage for all dependent variables was found to be less than 5%. The study’s results have provided evidence for the validity of the final models [62,63]. The cubic graph in S4 Fig illustrates the anticipated outcomes of the optimized formula, showcasing the normalized comparative impacts of the design parameters on the response variables.

**Table 4:** The composition and validation of the optimized formula with its predicted responses according to I Optimal Design.

Responses	Predicted value	Experimental value	% Relative error
EE%	76.4851 ± 3.21	80.01 ± 5.22	4.60
Vesicles size	543.201 ± 6.89	551.85 ± 2.68	1.59
Zeta potential	−45.00 ± 4.25	−43.88 ± 8.24	2.48

EE: entrapment efficiency; % Relative error (Experimental–Predicted)/Experimental × 100.

Data are mean values (n = 3) ± SD.

### 3.3. Characterization of axitinib- PEG- spanlastics

#### 3.3.1. VS, ZP, and EE% analysis.

The adding of PEG triggered the VS to upsurge from 551.85 ± 9.44 to 563.42 ± 8.63 nm, and ZP shifted from – 43.88 ± 0.21 to – 46.44 ± 0.09 mV. This increase in zeta potential ascribed to that PEG can adsorb onto the surface of particles, creating a hydrophilic layer. This layer can alter the surface charge distribution, leading to an increase in zeta potential. Moreover, PEG provides steric stabilization by creating a physical barrier around the particles. This barrier prevents particles from coming too close to each other, reducing the likelihood of aggregation and increasing the overall stability of the suspension [64]. Furthermore, the zeta potential of PEG-coated particles can also be influenced by the pH and ionic strength of the surrounding medium. PEG can help maintain a stable zeta potential across different pH levels and ionic strengths. The presence of PEG increased the VS, suggesting the successful production of axitinib- PEG- spanlastics. The EE% of axitinib within axitinib- PEG- spanlastics (84.32 ± 3.64%) was higher than that of axitinib- spanlastics (80.01 ± 3.06%). This phenomenon could potentially be ascribed to the enhanced solvent egress from the nanoparticles and the improved surface presentation of PEG molecules [65,66]. Also, PEG molecules may also act as a cosolvent, causing the drug to be soluble.

#### 3.3.2. Differential Scanning Calorimetry (DSC).

S5 Fig illustrates the thermograms obtained using differential scanning calorimetry (DSC) for span 60, SDC, axitinib, their physical mixture, and the optimized axitinib PEG-spanlastics. The endothermic peak of Span 60 occurs at a temperature of 54.53 °C (S5A Fig), which corresponds to its transition temperature [67]. the thermograph of SDC exhibited an exothermic recrystallization peak at 170.90°C, followed by an endothermic peak at 231.40°C (S5B Fig). This peak is likely attributed to the elimination of water molecules. The confirmation of the crystallinity of the pure medication axitinib was achieved through the observation of a distinct endothermic peak at around 228 ⁰C, which serves as an indicator of its unique melting point (S5C Fig). The DSC thermogram of Span 60, SDC, PEG, and axitinib physical mixture exhibited a distinct endothermic peak exclusively associated with axitinib observed around 224 °C (S5D Fig). This observation suggests that there is no significant shift in the drug peak so no chemical interaction occurring between the axitinib and spanlastics components. Additionally, the optimized axitinib- PEG- spanlastics (S5E Fig) exhibited a full elimination of the endothermic peak associated with axitinib, so validating the successful encapsulation of the drug within the PEG-spanlastics.

#### 3.3.3. X-ray Diffraction (XRD) analysis.

The X-ray diffraction (XRD) spectra of span 60, SDC, axitinib, their physical mixture, and the optimized axitinib-PEG-spanlastics were displayed in S6 Fig. The X-ray diffraction (XRD) pattern of the pure axitinib medication, as depicted in S6C Fig, exhibited multiple prominent peaks at 2ε values of 8.8, 9.4°, 11.9°, 14.5°, 15.1°, 15.6°, 19.0°, 19.3°, 20.3°, 20.6°, 21.6°, 23.1°, 24.1°, 24.6°, 24.9°, and 26.0°. These peaks provide evidence supporting the drug’s crystalline structure, as confirmed by Ren et al. [68]. The physical combination of Span-60, SDC, and axitinib did not exhibit any distinct drug peak which might be due to conversion of drug from crystalline to amorphous state, while a distinct excipient peak was observed (S6D Fig). However, the X-ray diffraction (XRD) spectrum of the optimal axitinib-PEG-spanlastics (S6E Fig) showed the complete absence of the drug peak, with only a single prominent peak observed at approximately 22°. This spectacle is likely ascribed to the presence of a PEG coating. The findings demonstrated that axitinib was fully encapsulated within PEG-spanlastics vesicles.

#### 3.3.4. Transmission Electron Microscopy (TEM).

S7 Fig displays transmission electron microscopy (TEM) image of axitinib-PEG-spanlastic. The spanlastic vesicles exhibit homogeneity, possession of well-defined boundaries, and have distinct spherical characteristics. The size achieved by transmission electron microscopy (TEM) is smaller than that obtained by dynamic light scattering using a Zetasizer Nano ZS (Malvern Instrument) due to the different analysis principles involved in each technique. The observed phenomenon can be attributed to the amphoteric properties of non-ionic surfactants, where in the hydrophobic component is positioned in a direction opposite to the aqueous milieu, while the hydrophilic component remains in touch with it. Furthermore, the inclusion of PEG resulted in the formation of almost spherical particles, albeit with some deformation, indicating a reduced rigidity in the nanoparticle structure. A disparity in density can be observed in the vicinity of the nanoparticles’ surface. The primary factor contributing to this observation is the existence of surface PEG molecules. In a more exact manner, the presence of surface PEG would have led to a dense brush confirmation. Previous studies have documented comparable findings with PEG-PLGA nanoparticles [69].

#### 3.3.5. *In vitro* drug release.

S8 Fig displays the in vitro release profile of axitinib from the PEG-spanlastic in comparison to the pure axitinib suspension. According to the data presented in S8 Fig, a drug release of 18.2 ± 2.72% occurred within the initial two-hour period of the investigation. This release was thereafter followed by sustained release. This attributed to that PEG-spanlastic systems provide sustained release by utilizing the swelling properties of PEG and other polymers, which control the rate at which the drug is released into the body [70].

The rapid release of the drug from the PEG-spanlastic material can be attributed to the adsorption of the drug onto the surface of the PEG polymer. In the investigation, it was shown that the PEG-spanlastic material demonstrated a significant increase in the cumulative release of axitinib (73.58 ± 3.37%) compared to the pure axitinib suspension (28.4 ± 4.38%) over a period of 4 hours. A statistically significant difference (p < 0.05) was seen in the release of axitinib-spanlastic compared to pure axitinib suspension. Table 5 shows the kinetic analysis of release data of the optimized formula compared to pure drug suspension. The release mechanism is dependent on the value of correlation coefficient. The release of Axitinib from both formulations was found to follow first order kinetics. This means that axitinib release is concentration dependent.

**Table 5:** The kinetic release data of the optimized formula compared to drug suspension.

Formula	Correlation Coefficient R^2^ According to			
	Zero	First	Second	Higuchi
Optimized axitinib-PEG-spanlastics	0.9354	0.9724	0.9720	0.949498
Axitinib suspension	0.9645	0.9701	0.9699	0.948592

#### 3.3.6. Stability studies.

The improved axitinib-PEG-spanlastic formulation exhibited a milky appearance and did not display any signs of separation, aggregation, or precipitation after being stored for a duration of one month at a temperature of 4 °C. Furthermore, it can be observed from S9 Fig that the alterations in size, entrapment, or surface charge exhibit negligible effects during the duration of storage. The high stability of axitinib- PEG -spanlastic may be attributed to their high ZP (− 42.65).

#### 3.3.7. Cytotoxic assay against t breast cancer cell line MCF-7 and ovarian cancer cell line OV-2774.

***3.3.7.1. Cell proliferation assay, WST-1*:** We have evaluated the optimized axitinib spanlastics, the optimized axitinib-PEG-spanlastics, and axitinib suspension for different dosages on two human cancer cell lines (MCF-7 and OV-2774). The growth inhibitory effects of axitinib spanlastics either with or without PEG were concentration-dependent in both cell lines (S10 Fig). The optimized axitinib spanlastics showed enhanced anticancer activity of axitinib on both cancer cell lines in comparison to axitinib suspension. The higher cellular uptake and regulated drug release of axitinib-spanlastics over the free drug may be responsible for their superior cytotoxic action [71,72]. Comparing spanlastics to the free drug, similar fascinating increases in medication efficacy have been previously discovered [73,74]. The inclusion of edge activators, also known as surfactants, in the spanlastics contributed to these effects. Surfactants are known to act as permeation enhancers and boost drug permeability through biological membranes. Furthermore, edge activators enhance the fluidity and deformability of the vesicle bilayer, facilitating their passage through the cell membrane, hence promoting drug accumulation within the cells [75].

The WST-1 assay revealed a reduction in cell viability percentage that was concentration dependant. (S10A Fig) showed a sharp reduction in the percent cell survival reaching 50% at a concentration of 0.68 µM for axitinib-PEG-spanlastics while the axitinib free drug solution showed 50% cell survival at 1.1 µM concentration in breast cancer (MCF-7) cell line. However, only the axitinib- PEG- spanlastics formula showed a 50% reduction in the cell survival at a concentration of 25 µM not axitinib free drug solution in ovarian cancer (OV-2774) cell line, (S10B Fig). This indicates that the axitinib-PEG-spanlastics formula was more effective in reducing 50% cell viability than the axitinib free drug suspension. This is explained by the high proportion of EE% and the presence of PEG coating, which promote the contact between the formula and the cell surface leading to the release of axitinib with potent intracellular absorption [76]. Furthermore, this could be related to the enhanced cellular uptake of axitinib-PEG-spanlastics formula in comparison to the axitinib free drug solution.

***3.3.7.2. Apoptosis*:** The percentages of apoptotic and necrotic cells in MCF-7 and OV-2774 cell lines (S11 Fig) were analyzed by flow cytometry. Incubation with either 0.68μM, or 25μM of axitinib-PEG-spanlastics nanoformula for 72 h significantly increased the percentage of apoptotic cells in MCF-7 and OV-2774 cells, respectively (S11A–S11C Fig). The % of apoptotic cells induced by axitinib-PEG-spanlastics relative to free drug solution was (70.76 ± 4.971% vs. 32.6 ± 1.803%) in MCF-7 and (43.55 ± 4.243% vs. 24.44 ± 4.950%) in OV-2774. This clearly shows that axitinib- PEG- spanlastics nanoformula is more effective than axitinib spanlastics and free axitinib in suppressing tumor growth and survival in human cancer cells and this may be due to PEG coating. These results complies with the previous research indicated that PEG surface coating on nanoparticles may enhance cellular absorption of nanoparticles and facilitate cell attachment [77].

#### 3.3.8. Molecular docking.

***3.3.8.1. Docking simulation*:** The docking experiments revealed that the predicted active sites of the tested receptors within the breast and ovarian cancer exhibited the highest binding affinity or scoring, as evidenced by a higher negative energy requirement. S12–S15 Figs. illustrate the process by which the Uni-prot server was employed to acquire the chemical structure and sequencing of the five proteins, as well as their recorded ID. The CB-Dock2 server was employed to identify the active sites of targeting for docking after the proteins were downloaded in pdb format. In addition to the shared amino acids, S16–S19 Figs. demonstrate that the figures included compartments. However, the grid box dimensions for each target protein were ultimately determined, as illustrated in S20–S23 Figs.

In spite of this, docking experiments demonstrated that the predicted active sites of the Axitinib and PEG ligand, as well as the identified protein receptors in the breast and ovarian receptors, respectively, exhibited the highest binding affinity. Axitinib and PEG exhibited a higher affinity binding to (Dopamine, VEGFR) and ovarian cancer (EGFR, BCL-xL) receptors affinity for Axitinib, with a value of (−8.0, −7.7) & (−2.8, −2.3) kcal/mol, respectively. Additionally, they exhibited a presence affinity for PEG (−2.9, −3.1) & (−1.5, −3.3) kcal/mol, respectively, for the same receptor types, which subsequently initiated controlled release for Axitinib (S24 and S25 Figs). The active site of a diverse array of breast and ovarian cancer proteins that were specifically targeted was investigated using molecular docking to determine the potential interaction mechanism between Axitinib and PEG.

The results of this study indicate that tested ligands and the targeted and selected proteins receptor on breast and ovarian cancer interact through a variety of hydrogen, conventional, covalent, and pi alkyl bonding interactions for both Axitinib and PEG. As the pathway of determination determined the interaction categories, the functional enrichment analysis revealed a number of interactions between overrepresented protein domains in the network. The presence of specific biological functions or pathways that are currently operational within the system may be suggested by this. Hydrophobic and H-bonding interactions are present in the preponderance of complexes. In comparison to Axitinib and PEG in 3D and 2D forms, S26–S33 Figs demonstrate the presence of H-bonding between the active side residues of the amide oxygen or nitrogen of the majority of compounds, albeit in a greater number of interactions.

Axitinib and PEG were assessed for their principal identical interactions with protein types in breast (Dopamine, VEGFR) and ovarian cancer (EGFR, BCL-xL) receptors. The primary mechanism of action for Axitinib and its coating with PEG for sustained release may be attributed to this binding affinity, in addition to other binding affinities to other proteins. According to the investigation’s results, Axitinib demonstrated a substantial affinity for the active sites of a diverse array of protein receptors that were identified. The EGFR binding receptor’s active site bound affinities for axitinib and PEG were −8.3% and −3.3 kcal/mol, respectively. Tayyab et al. [78] had previously confirmed the affinities for axitinib. Additionally, the same binding affinity was observed for another receptor. As shown by additional investigations of the interactions with the active sites, the ligand of active site 1 established alkyl bonds with the receptor over an average distance of angstroms. In organic molecules, alkyl interactions are typically weak and occur between homologous, nonreactive carbon groups. Pi-alkyl interactions between aromatic and aliphatic groups are distinguished by the intersection of the pi-electron density of the aromatic ring and the electron density of the alkyl group. Strong hydrophobic bonds, non-covalent van der Waals interactions, and H. bonds may account for a portion of the compound’s binding affinity, as suggested by these interactions. Feeble interactions known as hydrogen bonding are initiated by fluctuations in the electron density of atoms or molecules. The aforementioned interactions suggest that alpha guanine has the ability to form a stable complex with H. van der Waals forces and bonding.

The controlled release of a synergist compound to the anticancer activity of axitinib in a manner that is more efficacious and targeted to specific cancer receptors or proteins on both ovarian and breast cancer types is a mechanistic approach. In order to accomplish this objective, PEG-coated axitinib will enhance its anticancer activity, as demonstrated by our investigations and the dopamine-functionalized four-arm PEG, which enhances the efficacy and promotes the bioactivity and adhesive performance of Laponite drug [79]. In a previous study, it was also demonstrated that the VEGFR inhibitor axitinib’s inability to suppress tumor growth was a result of its involvement in the increase in the frequency of breast cancer stem cells. Additionally, the controlled enhancement of axitinib efficacy through the combination therapy of VEGFR inhibitors is a critical concern in the treatment of breast cancer [80,81]. Dopamine, a member of the catecholamine family, has been associated with the regulation of lactation and the development of the mammary organ in the field of breast physiology. Dopamine receptors are expressed in mammary epithelial cells, where they regulate the secretion of prolactin, a hormone that is essential for milk production. Furthermore, dopamine has been demonstrated to affect cell proliferation, differentiation, and apoptosis in the mammary gland, indicating a more extensive function in the maintenance of mammary tissue homeostasis [82–85]. Dopamine’s functionality is contingent upon its association with one of its five receptors, which are numbered D1 through D5. Additionally, the binding affinity of the receptors varies within their respective categories. Various aspects of breast cancer biology, such as cell proliferation, apoptosis, migration, invasion, and angiogenesis, have been associated with dysregulated dopaminergic signaling [86,87]. Ma et al. have previously confirmed the higher affinity and axitinib stimulation of dopamine receptors in breast cancer therapy and targeting [81].

Additionally, vascular endothelial growth factor receptor (VEGFR) inhibitors are employed in the treatment of breast cancer. Axitinib’s efficacy as a VEGFR inhibitor was previously studied; however, it is restricted to the treatment of breast cancer as a sole agent or in combination with other chemotherapeutic medications due to the potential for an increase in the number of cancer stem-like cells (CSCs) caused by axitinib. The current study assessed the efficacy of axitinib on MCF-7 breast cancer in vitro and molecular investigation in the presence of dopamine stimulation and VEGFR inhibitor [81].

Regarding the ovarian cancer receptors, the Epidermal Growth Factor Receptor (EGFR), a member of the Human Epidermal Receptor (HER) family, is highly expressed in a variety of malignancies, with ovarian cancer being the most prevalent [88]. It is a tyrosine kinase that is primarily activated by extracellular ligands that induce receptor autophosphorylation. This process may result in the activation of downstream pathways that are involved in angiogenesis, invasion, proliferation, and survival [89,90]. Recent research has demonstrated that the EGFR pathway is recurrently activated in the majority of cancer cells. The targeted inhibition of the pathway with a small molecule kinase inhibitor has been successful in the treatment of lung, breast, and ovarian cancer cells with EGFR mutations [91,92]. Throughout the past few decades, numerous studies have proposed the involvement of the EGFR pathway in the progression of cancer [92,93].

We discovered that the propensity of cancer cells to perish was increased as a result of the inhibition of mitochondrial protein BCL-xL in response to treatment with a wide range of oncology medications, such as chemotherapy and mitotic inhibitors [94]. The BCL-xL protein of breast cancer receptors is substantially inhibited by axitinib in this study due to its high binding affinity. This inhibition will be further enhanced by the controlled release of PEG. Regarding the role of BCL-xL inhibition, our docking results confirmed the findings of Nocquet et al. (2024) regarding other known ligands in cancer [95]. The string type of this interaction was accomplished by replacing this residue with specific amino acids, which increased the affinity of these compounds against the enzyme. Axitinib’s inhibition mechanisms are also believed to be influenced by hydrogen bonds with serine amino acids and π-cation and π-π interactions with tryptophan 86.

In the present investigation, the binding efficacy of axitinib and PEG, which have been experimentally bound to the receptors, is assessed by calculating binding affinity and binding free energy (ΔG). The binding site and interaction were meticulously illustrated, and an exhaustive search was conducted to identify the factors that improve the binding efficacy and enhance these interactions. Consequently, a comprehension of the factors that reinforce these interactions, in addition to their binding sites and interactions, facilitates the identification of potentially effective pharmaceuticals from the extensive pool of natural product and repurpose drug candidates. A computational chemistry, molecular docking, or in silico toxicity investigation can be highly beneficial in determining the reactivity of the ligand-receptor binding. Furthermore, the binding sites and mechanisms of action that were identified.

Subsequently, the objective of the molecular docking investigations conducted in this study was to precisely determine the mechanism of action and distinct pathway for the anticancer activity of the tested ligand. The objective of this investigation was to assess the in vitro efficacy of axitinib against both forms of cancer and to establish a correlation between this effect and the inhibition of critical proteins that are essential for the survival of cancer cells.

## 4. Conclusion

Axitinib-loaded PEG-spanlastics have been effectively developed for the purpose of targeting breast and ovarian cancer. The stability and encapsulation efficiency of axitinib-PEG-spanlastics were remarkably high. The release profile of the substances exhibited a burst release followed by a sustained release. Based on molecular docking, it is feasible to deduce that Axitinib and PEG diminished the activity of VEGDR, EGFR, and BCL-xL, while simultaneously stimulating the dopamine receptor, which in turn led to an increase in anticancer activity. As a result, Axitinib-PEG- spanlastics could be used for the effective treatment of ovarian and breast malignancies.

## Supporting information

S1 Fig 3D Response surface plot and cube graph for the effect of independent variables on the dependent responses, (A) EE%, (B) Vesicles size, and (C) Zeta potential. (PDF)

S2 Fig Contour plot for the effect of independent factors on different responses. (PDF)

S3 Fig The composition of the optimized formula with its expected responses according to I Optimal Design. (PDF)

S4 Fig Cube graph for the expected responses of the optimized formula with its desirability. (PDF)

S5 Fig DSC thermograms of (A) Span 60; (B) SDC; (C) Axitinib; (D) Span 60, SDC, PEG, and Axitinib physical mixture; (E) the optimized axitinib-PEG-spanlastics. (PDF)

S6 Fig XRD patterns of (A) Span 60; (B) SDC; (C) Axitinib; (D) Span 60, SDC, PEG, and Axitinib physical mixture; (E) the optimized axitinib-PEG-spanlastics. (PDF)

S7 Fig TEM image of the optimized axitinib-PEG-spanlastics. (PDF)

S8 Fig In-vitro release of Axitinib from the optimized formula compared to drug suspension. (PDF)

S9 Fig The effect of storage on the optimized formula. (PDF)

S10 Fig Cytotoxicity of the optimized axitinib-PEG-spanlastics compared to the optimized axitinib spanlastics, axitinib suspension, and plain spanlastics in cancer cells. (A) MCF-7 cells were treated with 0.4-fold serial dilution increase in all axitinib formulations (0.4 µM–1.6 μM). (B) OV-2774 cells were treated with 10-fold serial dilution increase in all axitinib spanlastics nano formulations (10 µM–40 μM). The WST-1 assay was used to investigate the effects of axitinib in different formulations on the cell viability. Cell viability was expressed as a percentage of live cells relative to 0 µm of the treatment. Medications showed a concentration-dependent reduction in cell viability. The comparisons between groups were analyzed using one way analysis of variance (ANOVA). Analysis was performed using GraphPad Prism 9. Results were expressed as mean ± standard deviation (SD). * *p* < 0.05 versus free drug.
(PDF)

S11 Fig The Effect of Axitinib spanlastics and Axitinib-PEG-spanlastics on cancer apoptosis induction. (A) MCF-7 cells were treated with (1.1 μM) for both plain spanlastics and axitinib suspension, (1 μM) for axitinib spanlastics and (0.68 μM) for axitinib-PEG-spanlastics for 72 h. And (B) OV-2774 cells were treated with (40 μM) for both plain and free drug, (30 μM) for axitinib spanlastics and (25 μM) for axitinib-PEG-spanlastics for 72 hrs. Treated cells were stained with Annexin V and PI and analyzed by flow cytometry. (C) The percentage of apoptotic cells was significantly higher in axitinib-PEG-spanlastics compared to drug suspension (70.76% vs. 32.6%) in MCF-7 and (43.55 vs 24.44) in OV-2774. The comparisons between groups were analyzed using one way analysis of variance (ANOVA). Analysis was performed using Microsoft Office Excel 2016 and GraphPad Prism 9. Results were expressed as mean ± standard deviation (SD). * *p* < 0.05, ** *p* < 0.01 and *** *p* < 0.001 versus free drug.
(PDF)

S12 Fig Dopamine Receptor.pdb.id & chemical structure (6 CM4) Structure, ID code and origin or source of protein with the shared amino acids. (PDF)

S13 Fig VEGF Receptor.pdb.id & chemical structure (3V2A) Structure, ID code and origin or source of protein with the shared amino acids. (PDF)

S14 Fig EGFR Receptor.pdb.id & chemical structure (5WB7) Structure, ID code and origin or source of protein with the shared amino acids. (PDF)

S15 Fig BCL-xL Receptor.pdb.id & chemical structure (1YSG) Structure, ID code and origin or source of protein with the shared amino acids. (PDF)

S16 Fig Pocket, target of Dopamine protein and its shared amino acids in the active site of binding with axitinib. (PDF)

S17 Fig Pocket, target of VEGFR protein and its shared amino acids in the active site of binding with axitinib. (PDF)

S18 Fig Pocket, target of EGFR protein and its shared amino acids in the active site of binding with axitinib. (PDF)

S19 Fig Pocket, target of BCL-xL protein and its shared amino acids in the active site of binding with axitinib. (PDF)

S20 Fig Grid box centers and dimensions for dopamine protein, for both ligands Axitinib and PEG. (PDF)

S21 Fig Grid box centers and dimensions for VEGFR protein, for both ligands Axitinib and PEG. (PDF)

S22 Fig Grid box centers and dimensions for BCL-xL protein, for both ligands Axitinib and PEG. (PDF)

S23 Fig Grid box centers and dimensions for EGFR protein, for both ligands Axitinib and PEG. (PDF)

S24 Fig Binding affinity (kcal/mol) scoring degree of binding and interactions between Axitinib& PEG on dopamine and VEGFR receptors. (PDF)

S25 Fig Binding affinity (kcal/mol) scoring degree of binding and interactions between Axitinib& PEG on EGFR & BCL-xL receptors. (PDF)

S26 Fig 3D &2D docking interactions sites between Axitinib and Dopamine. (PDF)

S27 Fig 3D &2D docking interactions sites between PEG and Dopamine. (PDF)

S28 Fig 3D &2D docking interactions sites between Axitinib and VEGFR. (PDF)

S29 Fig 3D &2D docking interactions sites between PEG and VEGFR. (PDF)

S30 Fig 3D &2D docking interactions sites between Axitinib and EGFR. (PDF)

S31 Fig 3D &2D docking interactions sites between PEG and EGFR. (PDF)

S32 Fig 3D &2D docking interactions sites between Axitinib and BCL-xL. (PDF)

S33 Fig 3D &2D docking interactions sites between PEG and BCL-xL. (PDF)

S1 Appendix (DOCX)

S1 File Original data_MCF7 cell lines. (PDF)

S2 File Original data_OV-2774 cell lines. (PDF)

S3 File Supporting file. (DOCX)
