# Supplementary figures and images for: Fabrication and appraisal of axitinib loaded PEGylated spanlastics against MCF- 7 and OV- 2774 cell lines using molecular docking methods and in-vitro study
Source: PLoS One. 2025 Jul 1;20(7):e0325055. doi: 10.1371/journal.pone.0325055 (PMC12212535; doi:10.1371/journal.pone.0325055)

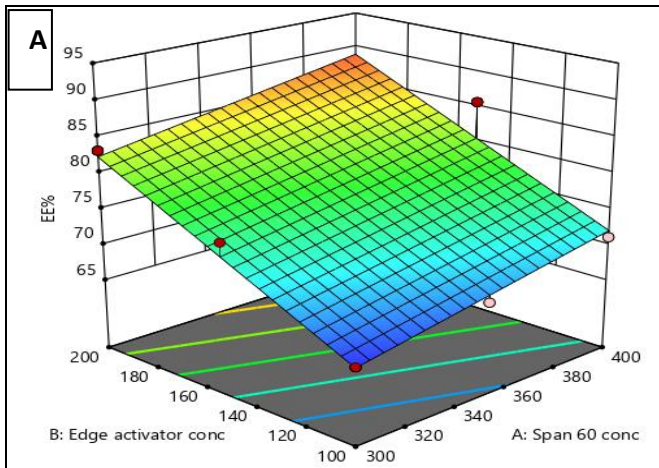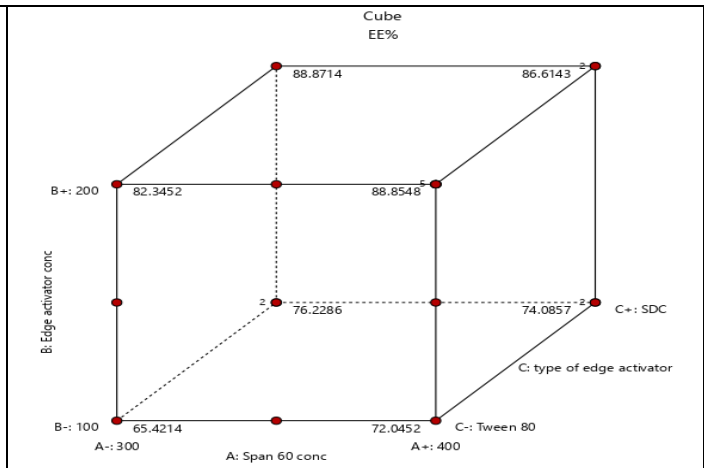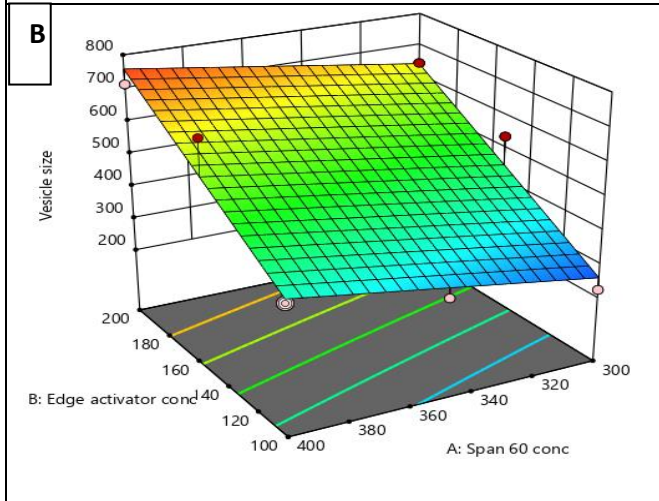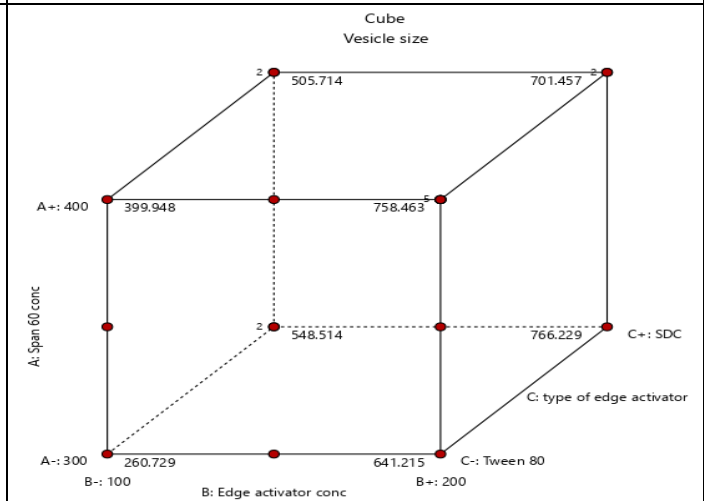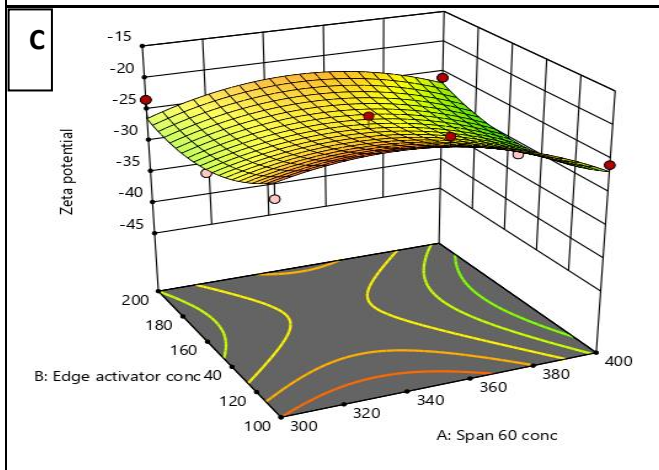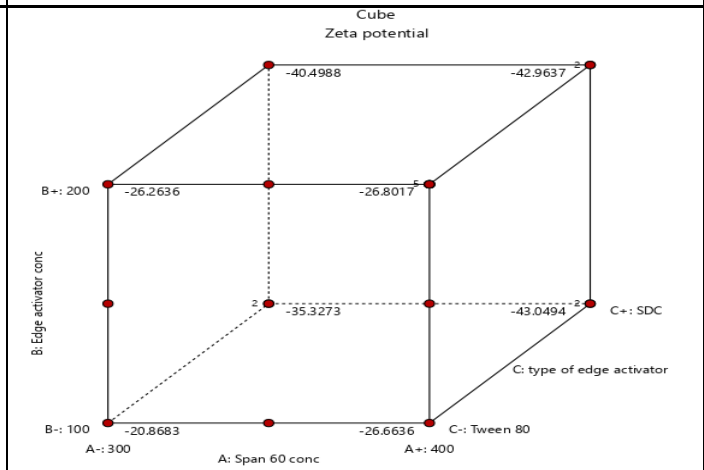

Supplement: S1 Fig — (PDF) [file pone.0325055.s001.pdf]

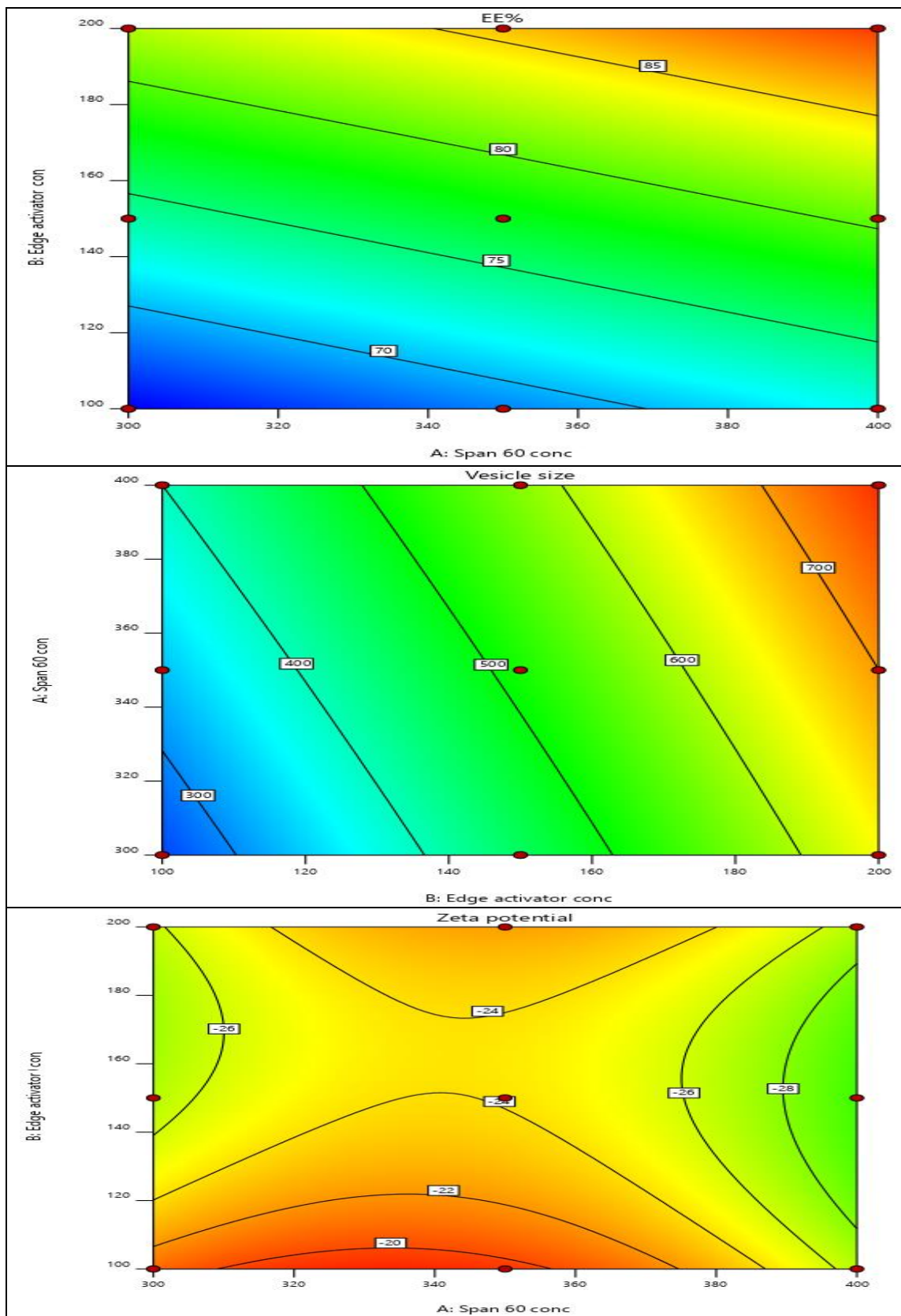

Supplement: S2 Fig — (PDF) [file pone.0325055.s002.pdf]

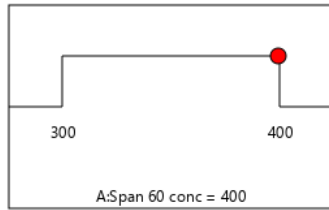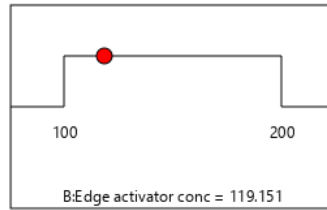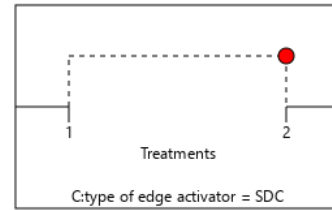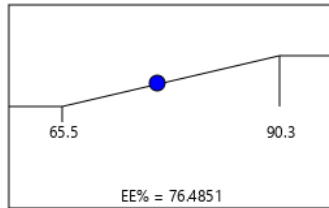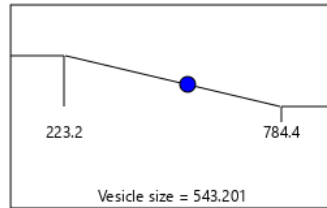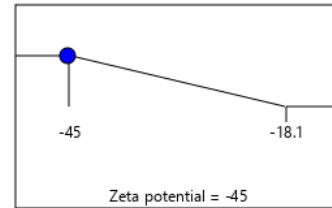

Desirability = 0.731  
Solution 1 out of 20

Supplement: S3 Fig — (PDF) [file pone.0325055.s003.pdf]

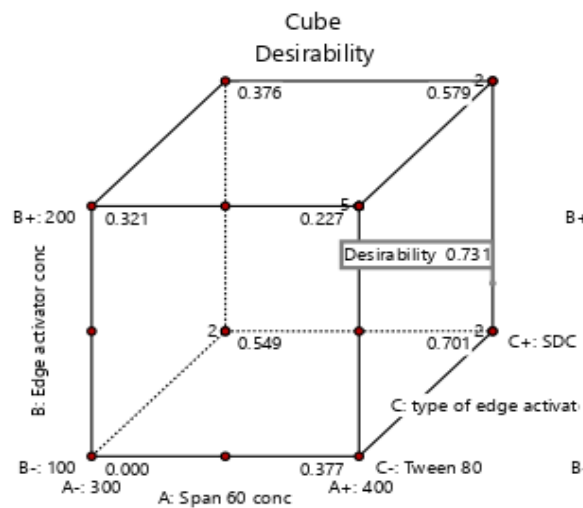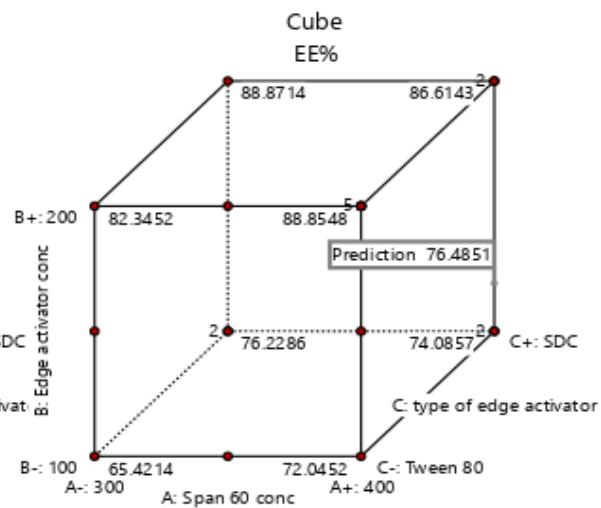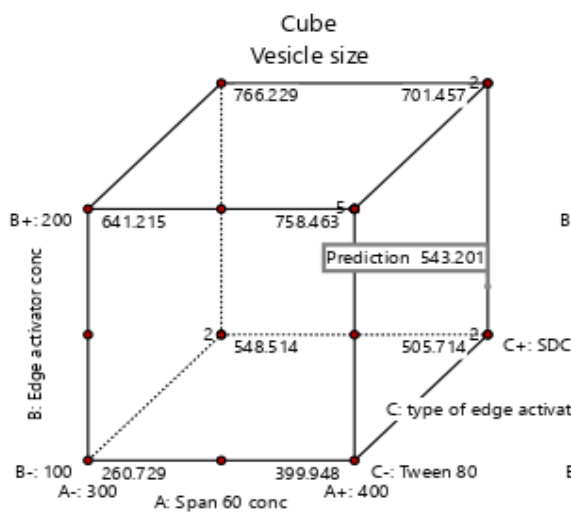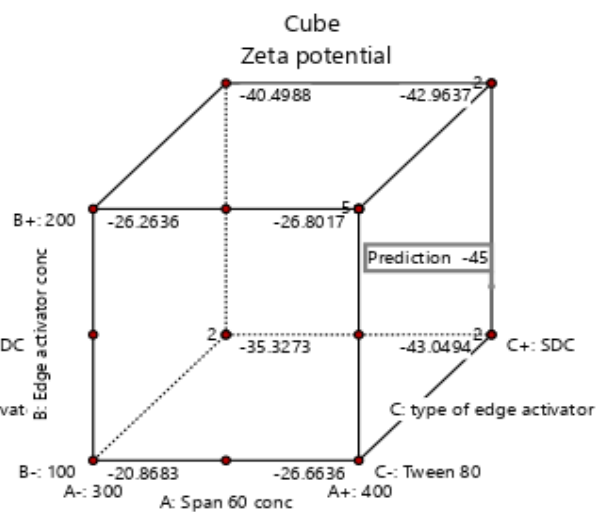

Supplement: S4 Fig — (PDF) [file pone.0325055.s004.pdf]

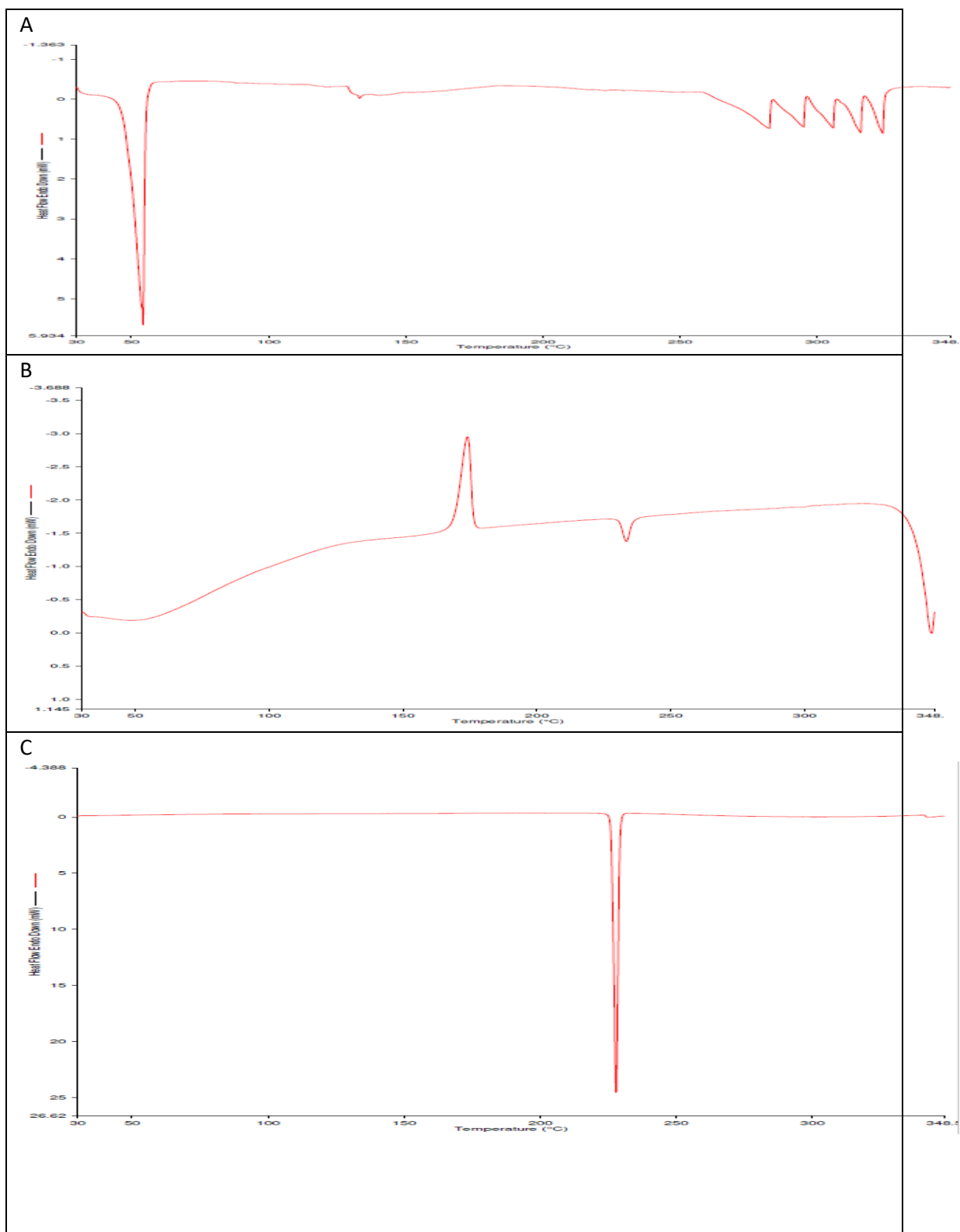

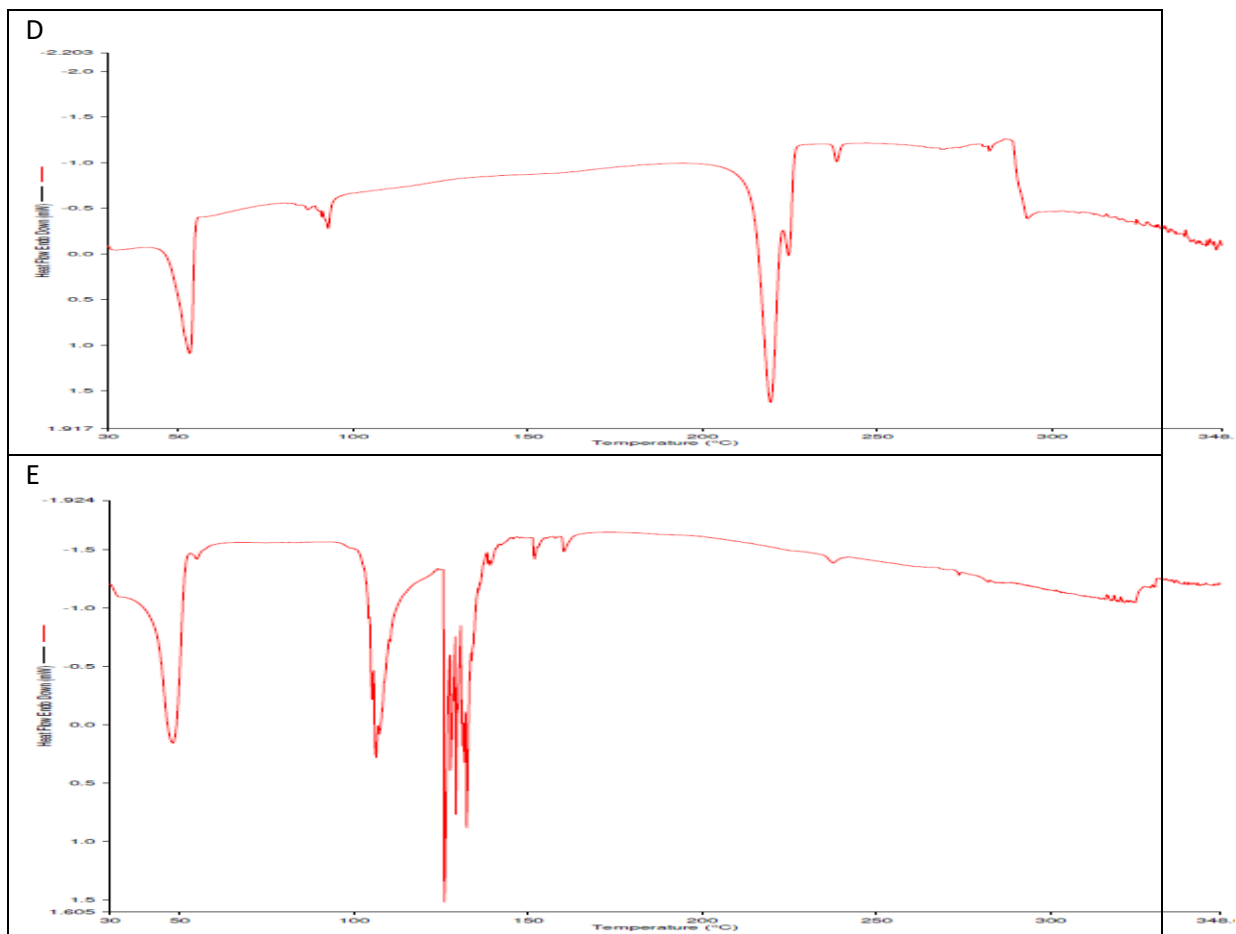

Supplement: S5 Fig — (PDF) [file pone.0325055.s005.pdf]

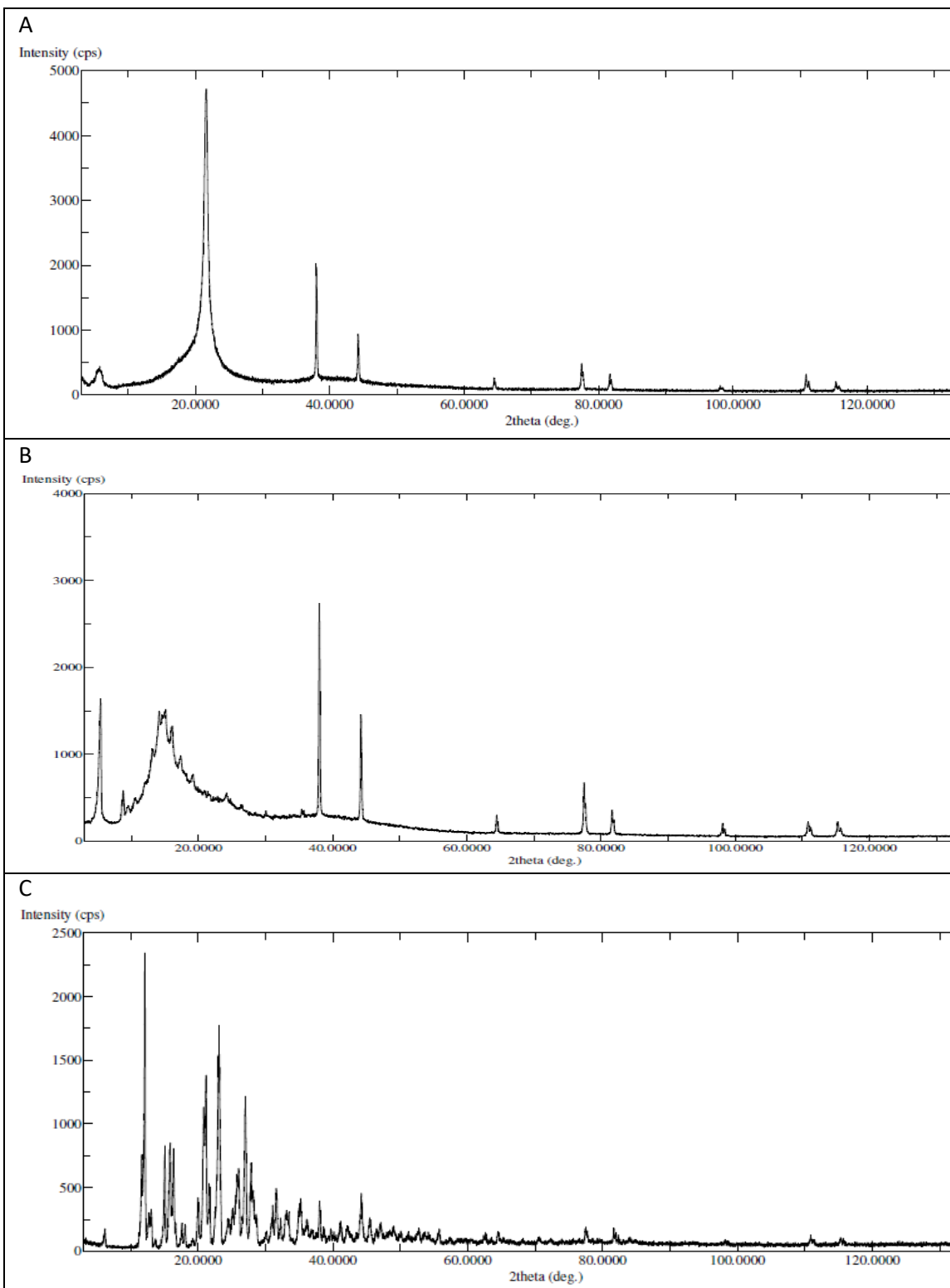

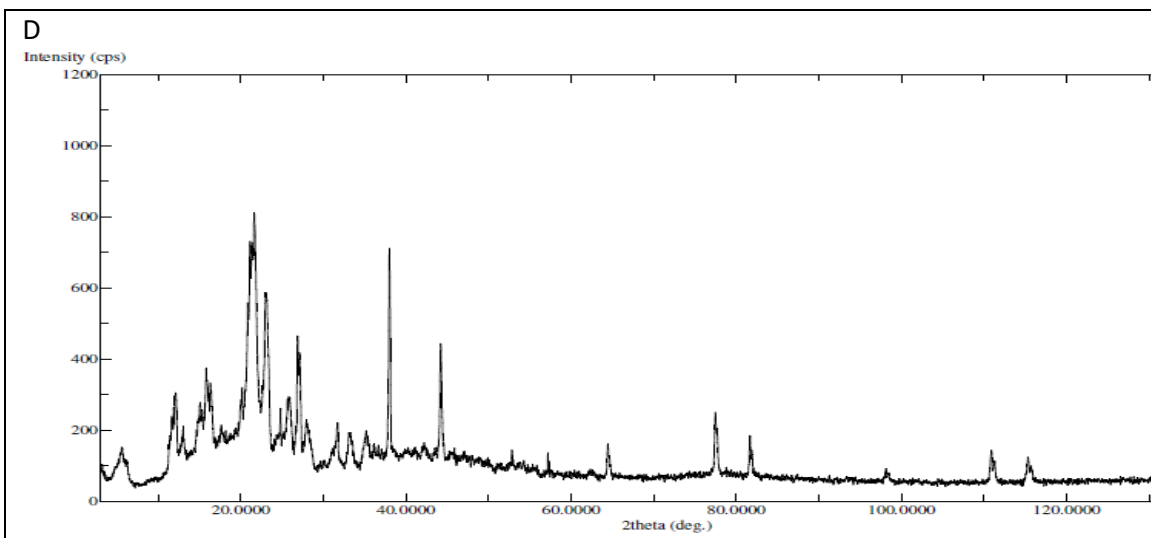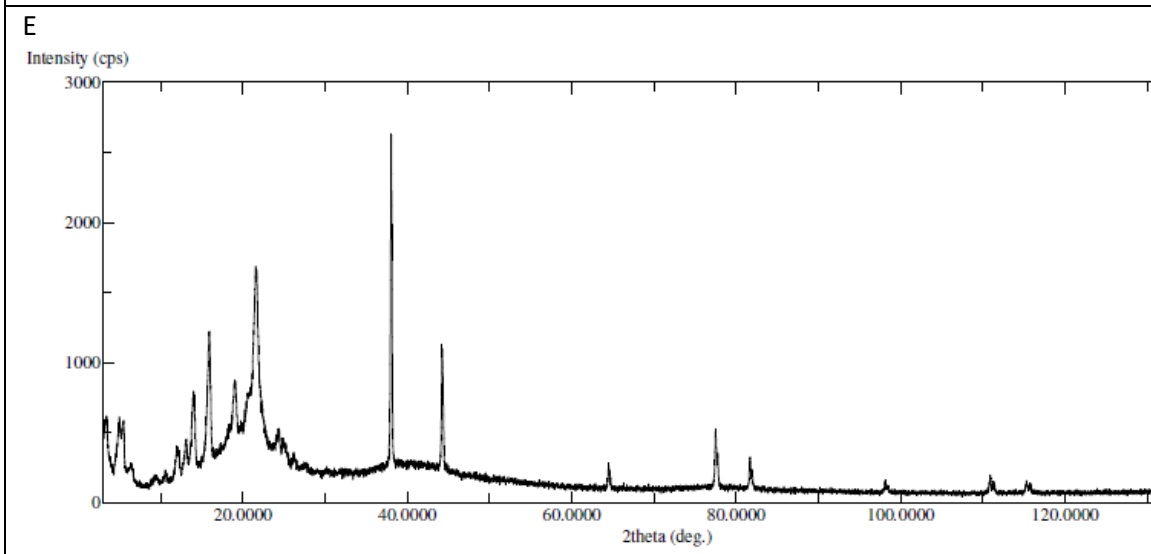

Supplement: S6 Fig — (PDF) [file pone.0325055.s006.pdf]

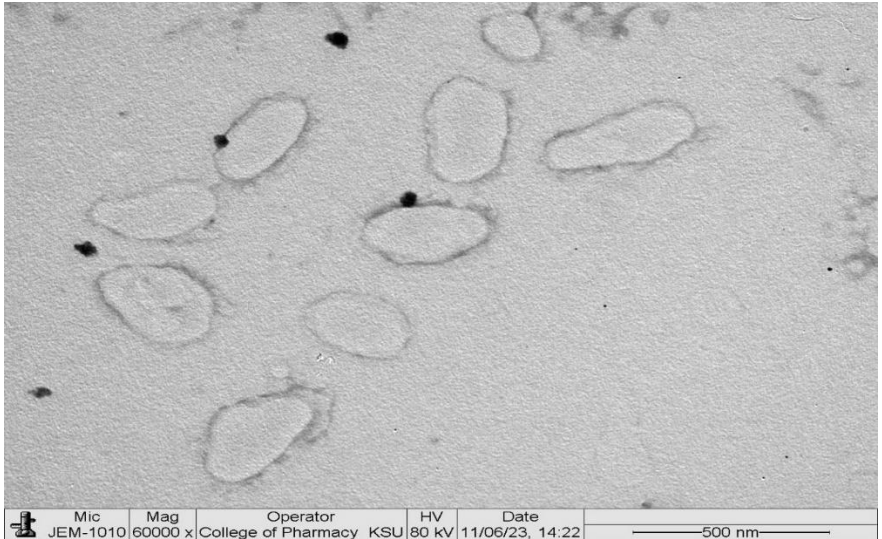

|                                                                                   |          |         |                         |       |                 |        |
|-----------------------------------------------------------------------------------|----------|---------|-------------------------|-------|-----------------|--------|
| 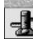 | Mic      | Mag     | Operator                | HV    | Date            |        |
| JEM-1010                                                                          | JEM-1010 | 60000 x | College of Pharmacy KSU | 80 kV | 11/06/23, 14:22 | 500 nm |

Supplement: S7 Fig — (PDF) [file pone.0325055.s007.pdf]

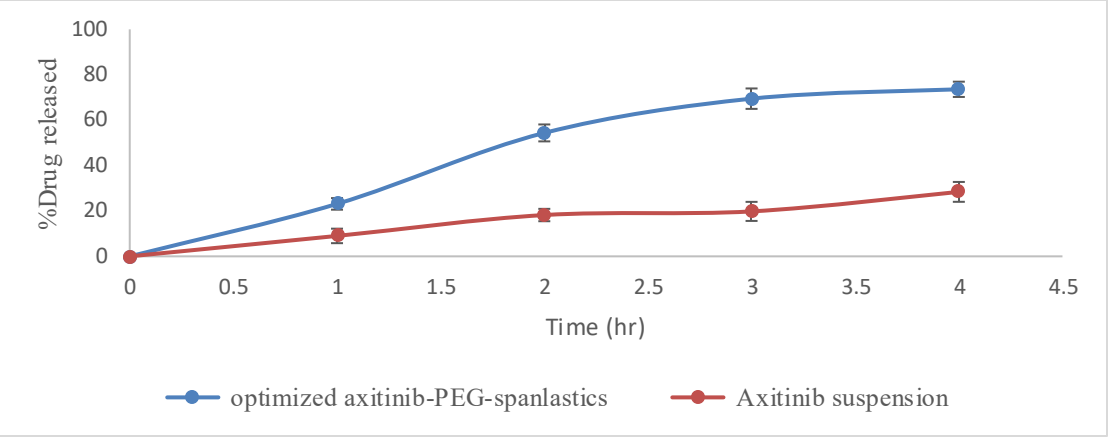

Supplement: S8 Fig — (PDF) [file pone.0325055.s008.pdf]

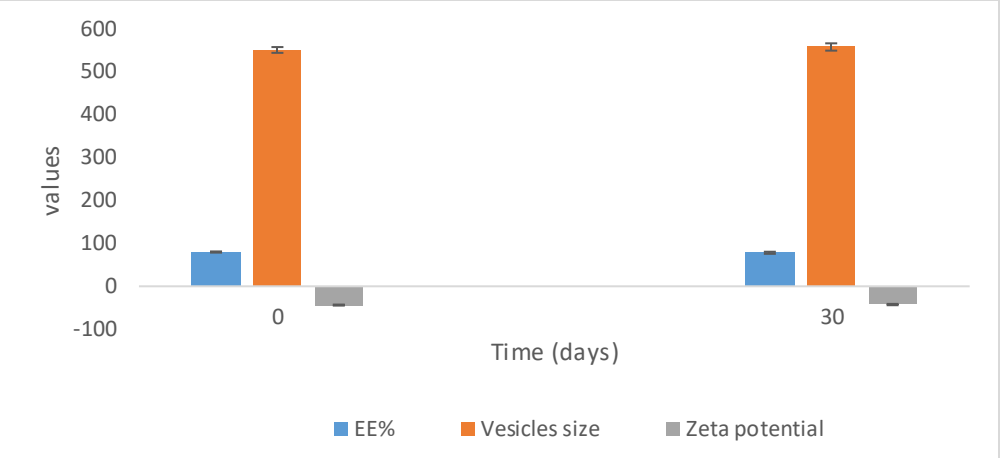

Supplement: S9 Fig — (PDF) [file pone.0325055.s009.pdf]

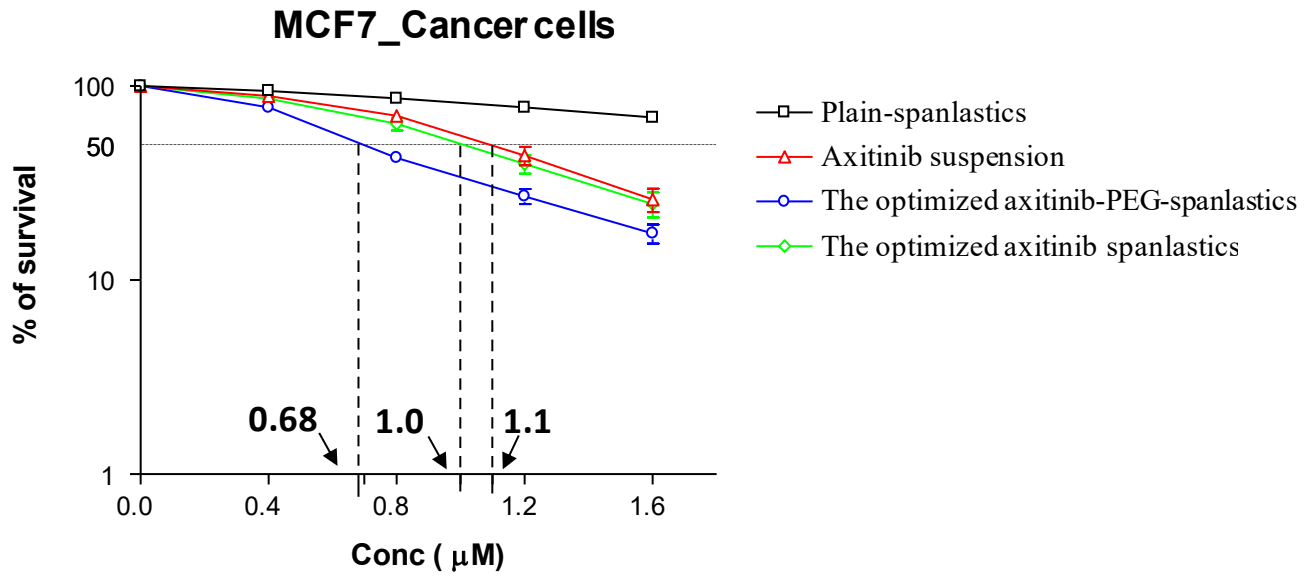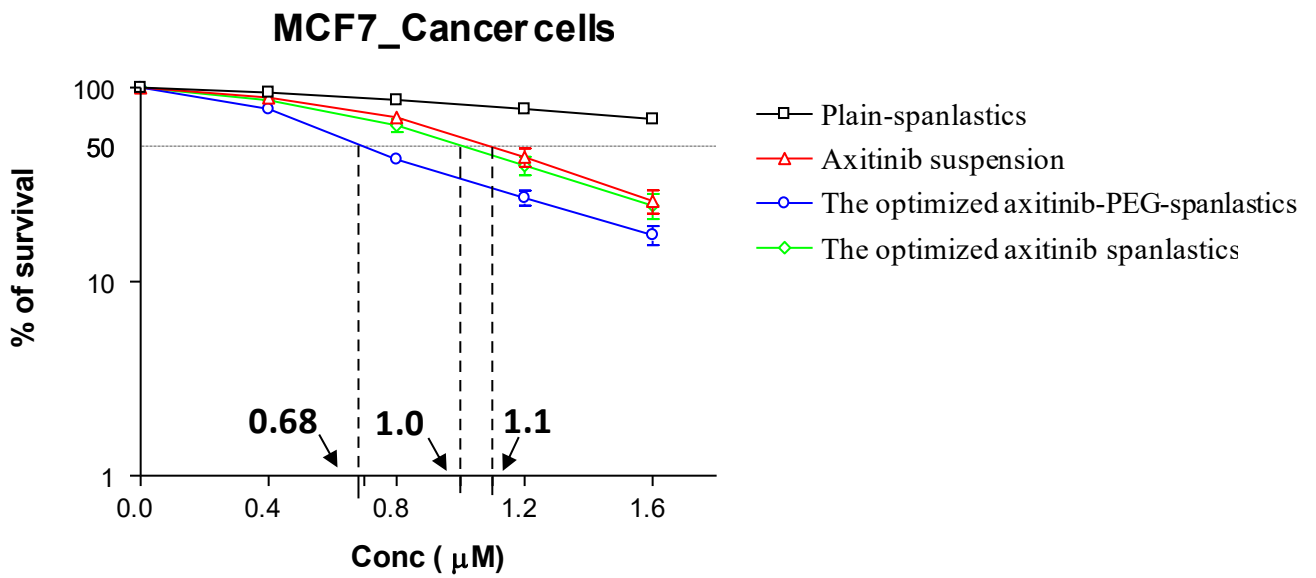

Supplement: S10 Fig — (A) MCF-7 cells were treated with 0.4-fold serial dilution increase in all axitinib formulations (0.4 µM–1.6 μM). (B) OV-2774 cells were treated with 10-fold serial dilution increase in all axitinib spanlastics nano formulations (10 µM–40 μM). The WST-1 assay was used to investigate the effects of axitinib in different formulations on the cell viability. Cell viability was expressed as a percentage of live cells relative to 0 µm of the treatment. Medications showed a concentration-dependent reduction in cell viability. The comparisons between groups were analyzed using one way analysis of variance (ANOVA). Analysis was performed using GraphPad Prism 9. Results were expressed as mean ± standard deviation (SD). * p < 0.05 versus free drug. (PDF) [file pone.0325055.s010.pdf]

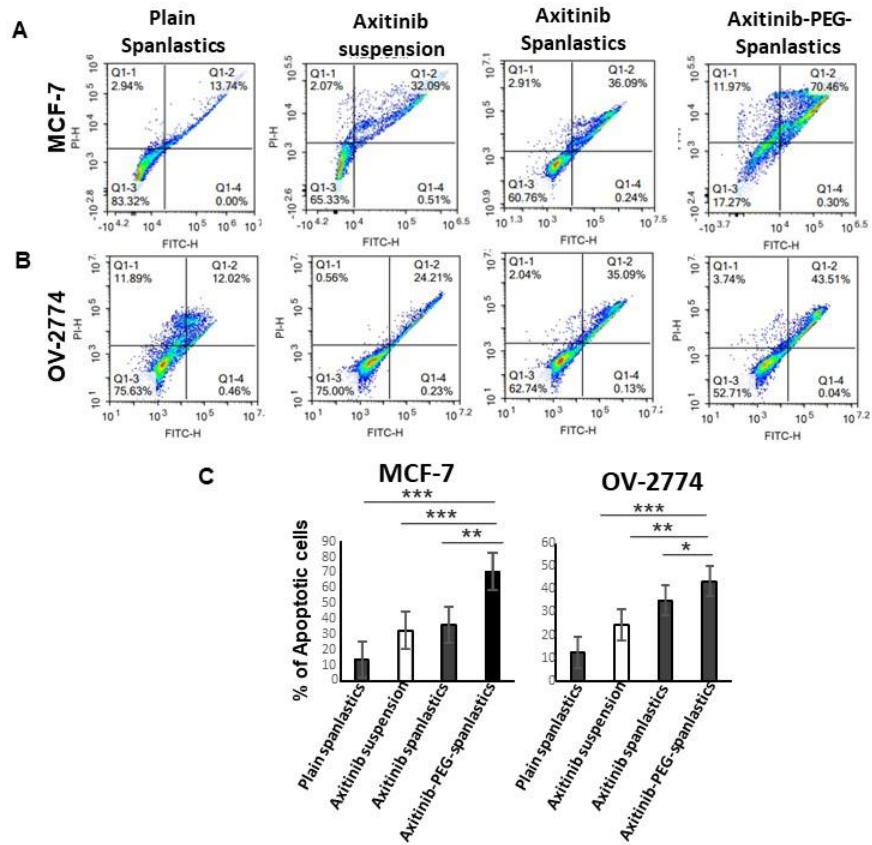

Supplement: S11 Fig — (A) MCF-7 cells were treated with (1.1 μM) for both plain spanlastics and axitinib suspension, (1 μM) for axitinib spanlastics and (0.68 μM) for axitinib-PEG-spanlastics for 72 h. And (B) OV-2774 cells were treated with (40 μM) for both plain and free drug, (30 μM) for axitinib spanlastics and (25 μM) for axitinib-PEG-spanlastics for 72 hrs. Treated cells were stained with Annexin V and PI and analyzed by flow cytometry. (C) The percentage of apoptotic cells was significantly higher in axitinib-PEG-spanlastics compared to drug suspension (70.76% vs. 32.6%) in MCF-7 and (43.55 vs 24.44) in OV-2774. The comparisons between groups were analyzed using one way analysis of variance (ANOVA). Analysis was performed using Microsoft Office Excel 2016 and GraphPad Prism 9. Results were expressed as mean ± standard deviation (SD). * p < 0.05, ** p < 0.01 and *** p < 0.001 versus free drug. (PDF) [file pone.0325055.s011.pdf]

# Dopamine -Receptor

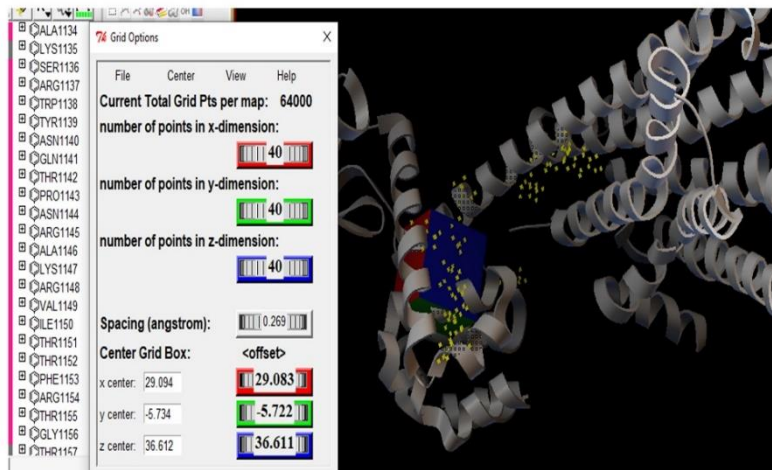

*Grid box dimensions , active sites*

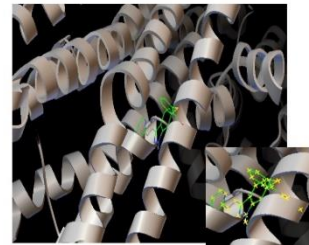

**Axitinib**

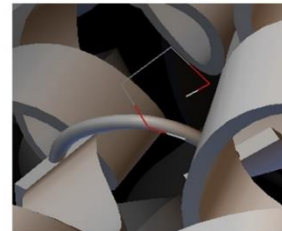

**PEG**

Supplement: S20 Fig — (PDF) [file pone.0325055.s020.pdf]

# VEGFR-Receptor

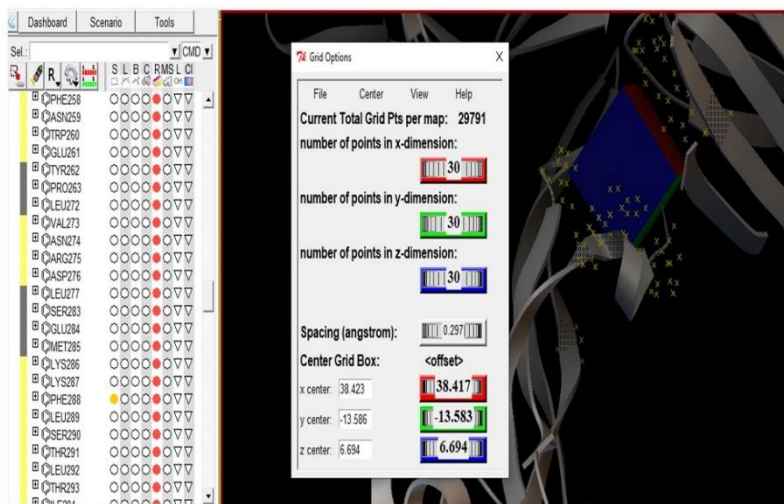

*Grid box dimensions , active sites*

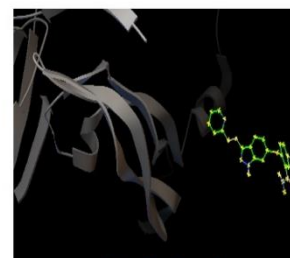

**Axitinib**

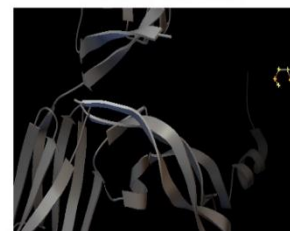

**PEG**

Supplement: S21 Fig — (PDF) [file pone.0325055.s021.pdf]

# BCL-xL-Receptor

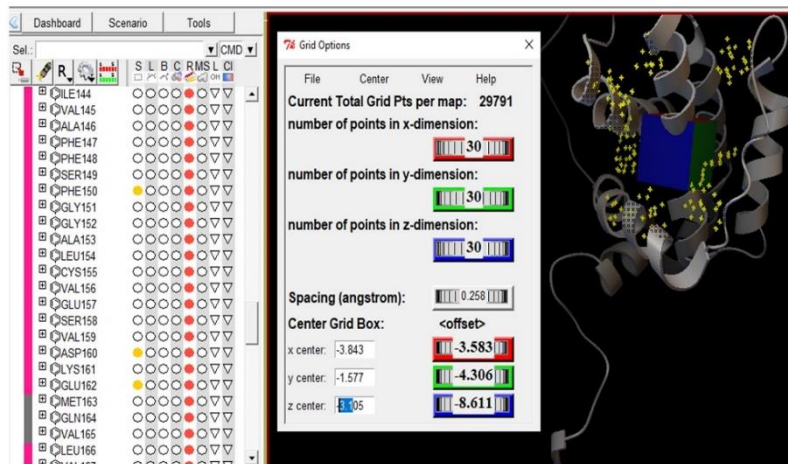

*Grid box dimensions , active sites*

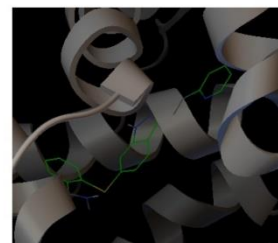

**Axitinib**

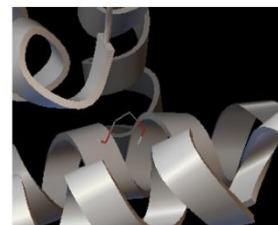

**PEG**

Supplement: S22 Fig — (PDF) [file pone.0325055.s022.pdf]

# EGFR-Receptor

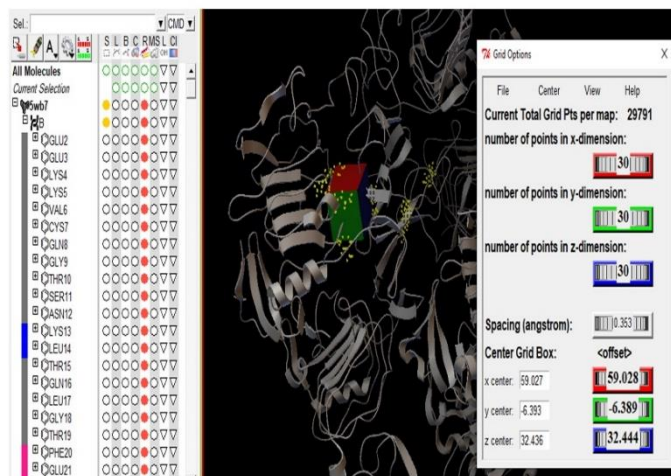

*Grid box dimensions , active sites*

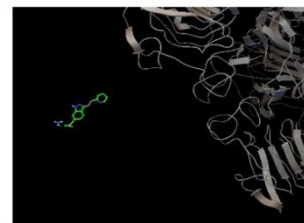

**Axitinib**

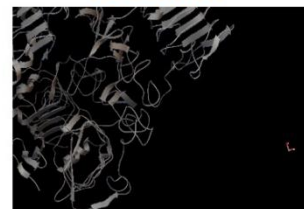

**PEG**

Supplement: S23 Fig — (PDF) [file pone.0325055.s023.pdf]

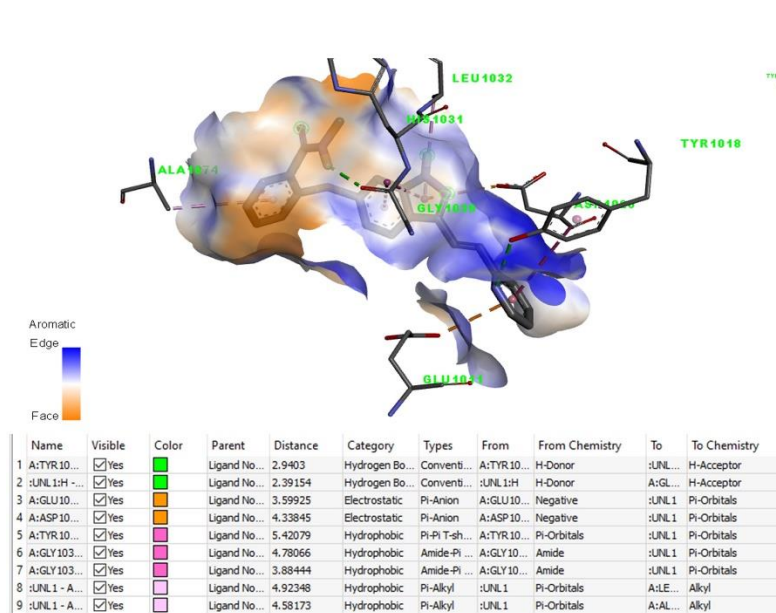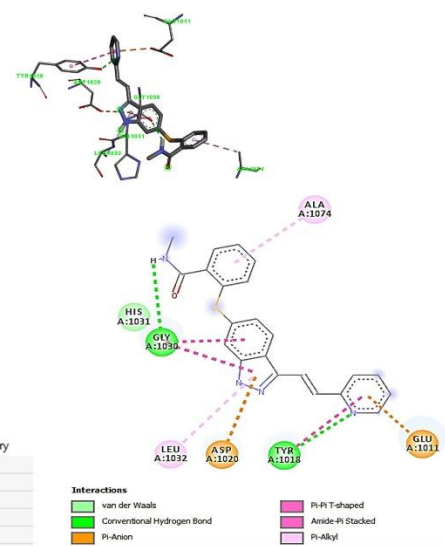

*2D interaction – Dopamine & Axitinib*

*3D interaction & shared Amino acids of Dopamine & Axitinib*

Supplement: S26 Fig — (PDF) [file pone.0325055.s026.pdf]

## VEGFR & PEG

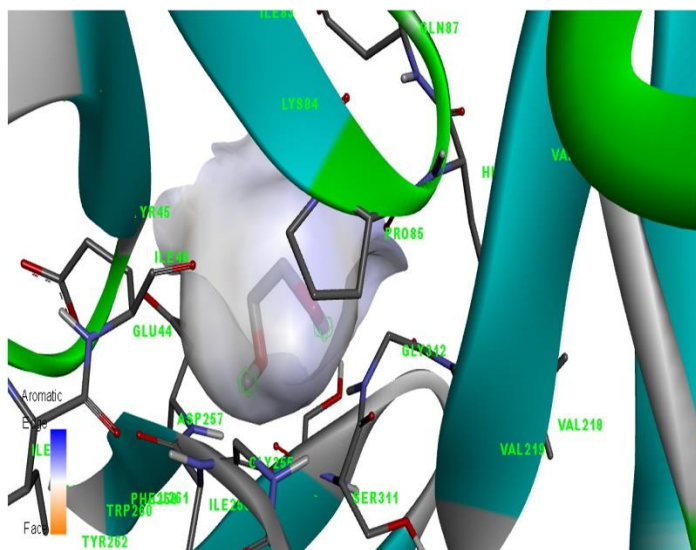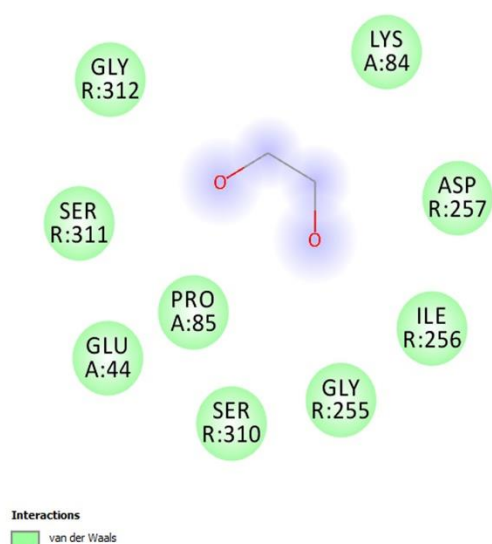

Supplement: S29 Fig — (PDF) [file pone.0325055.s029.pdf]

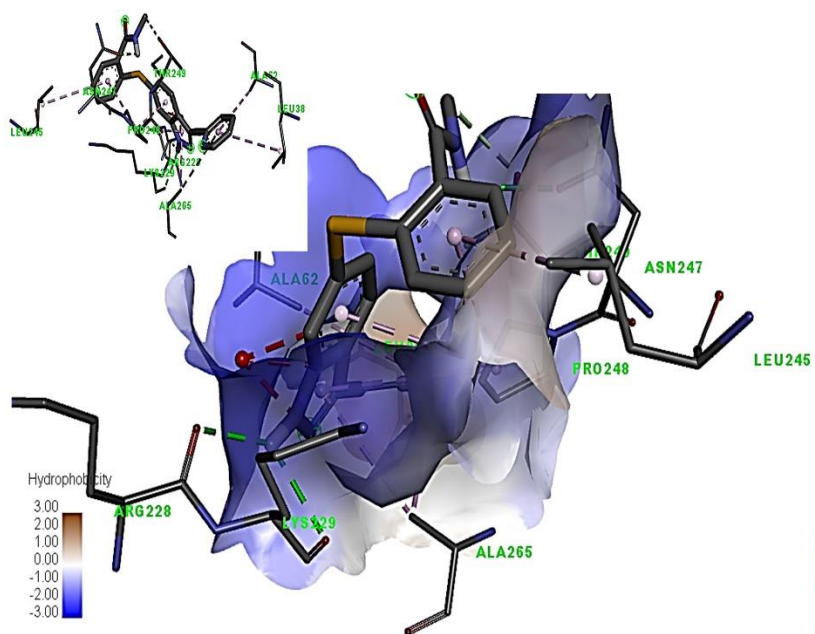

*3D interaction & shared Amino acids of EGFR & Axitinib*

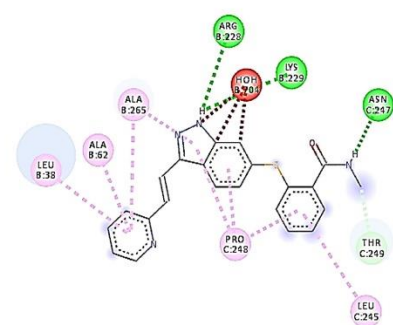

*2D interaction –EGFR & Axitinib*

Supplement: S30 Fig — (PDF) [file pone.0325055.s030.pdf]

## EGFR&PEG

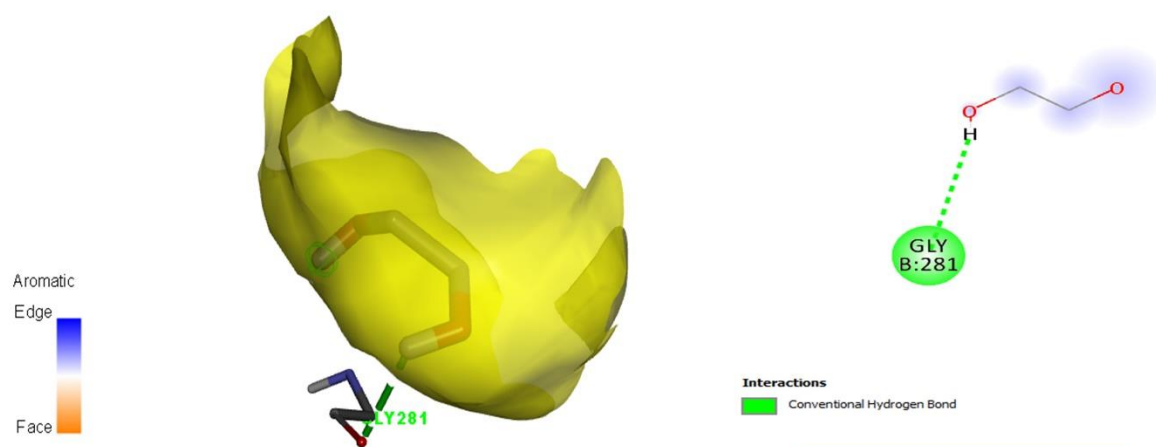

*2D interaction -EGFR & PEG*

*3D interaction & shared Amino acids of EGFR & PEG*

Supplement: S31 Fig — (PDF) [file pone.0325055.s031.pdf]

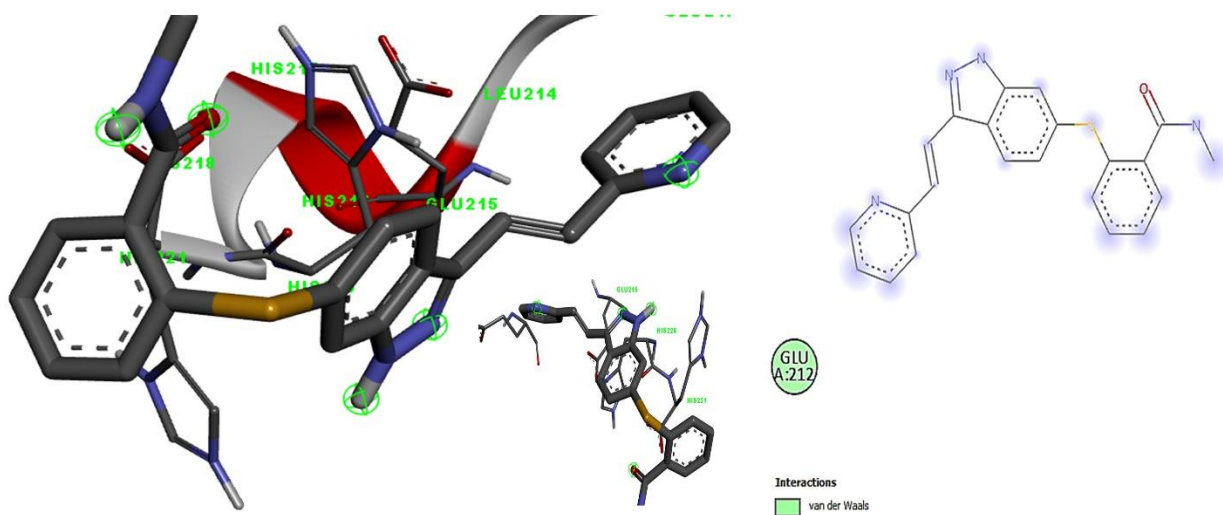

*3D interaction & shared Amino acids of BCL-xL & Axitinib*

*2D interaction –BCL-xL & Axitinib*

Supplement: S32 Fig — (PDF) [file pone.0325055.s032.pdf]

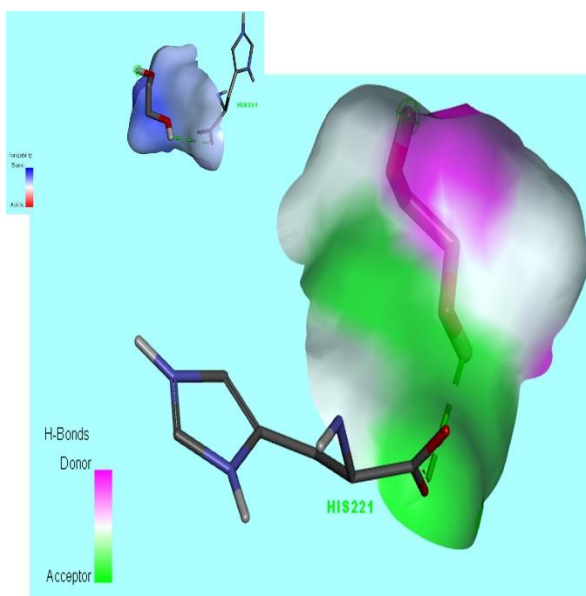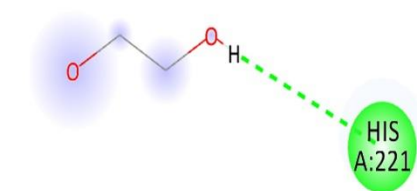

Interactions  
 Conventional Hydrogen Bond

Supplement: S33 Fig — (PDF) [file pone.0325055.s033.pdf]

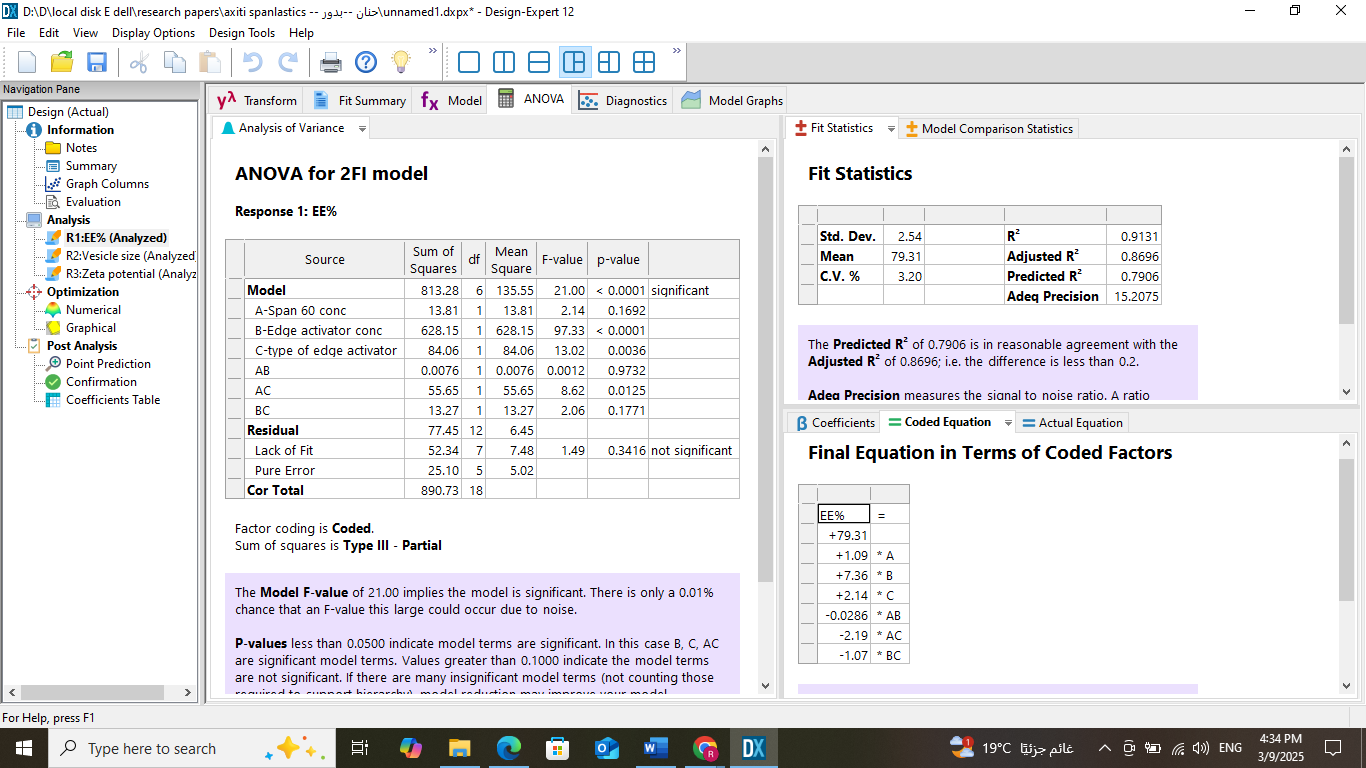


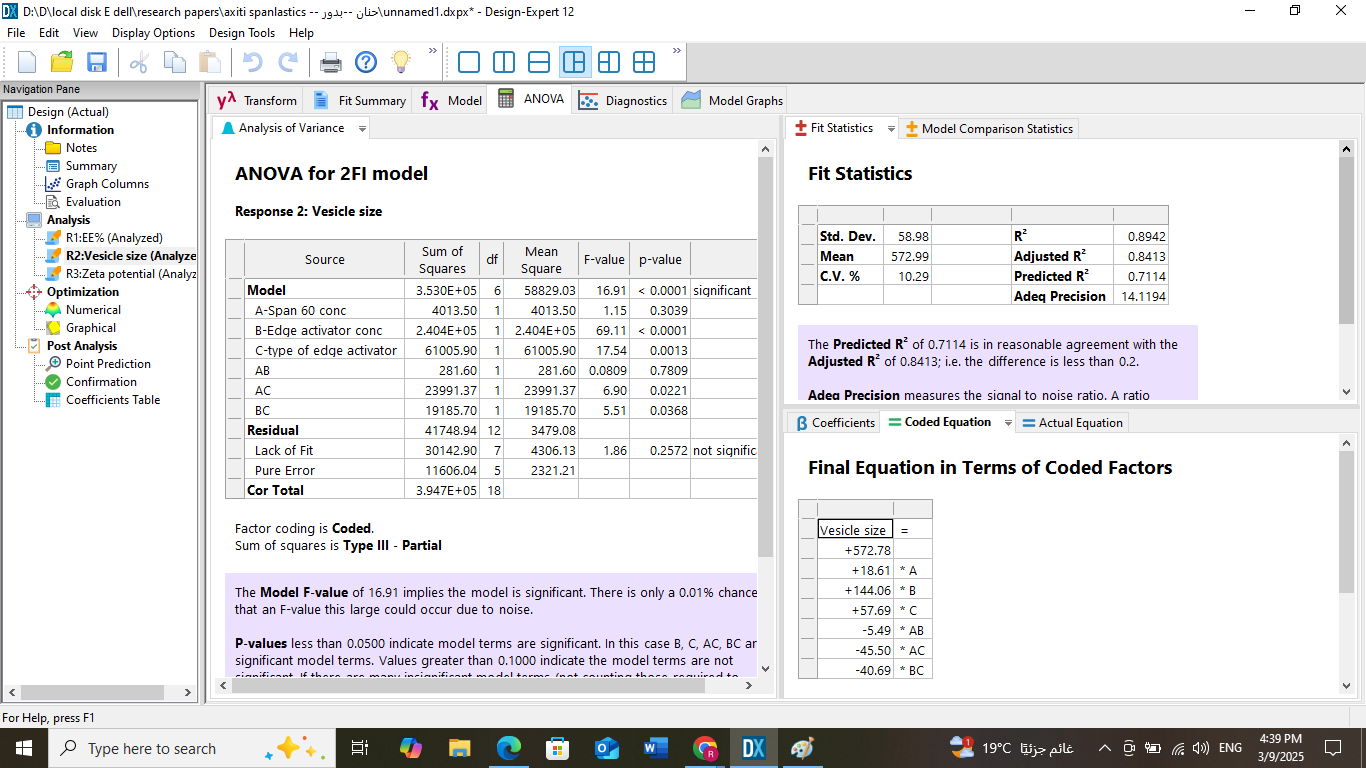


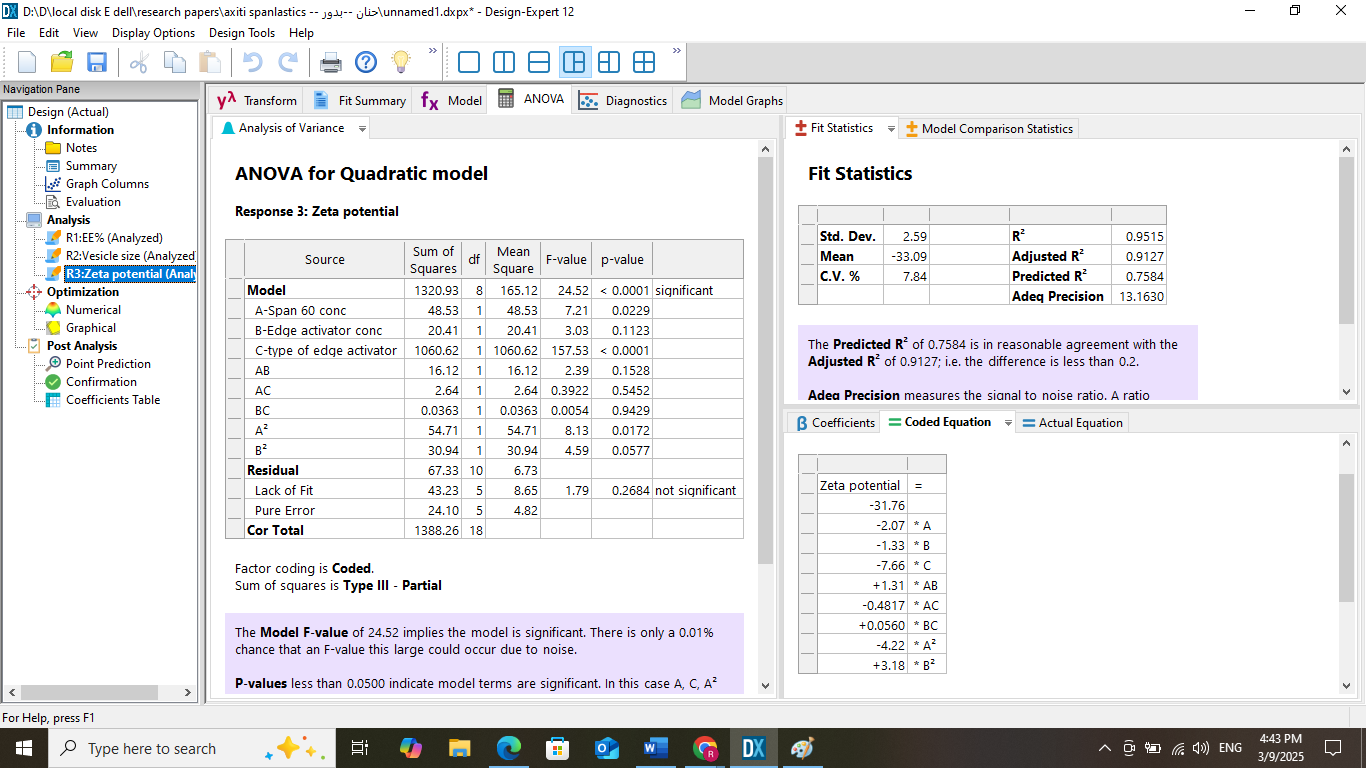

Supplement: S3 File — (DOCX) [file pone.0325055.s037.docx]
