# Supplementary material for: Fabrication and appraisal of axitinib loaded PEGylated spanlastics against MCF- 7 and OV- 2774 cell lines using molecular docking methods and in-vitro study
Source: PLoS One. 2025 Jul 1;20(7):e0325055. doi: 10.1371/journal.pone.0325055 (PMC12212535; doi:10.1371/journal.pone.0325055)
Supplement: S12 Fig — (PDF) [file pone.0325055.s012.pdf]

Dopamine/Receptor

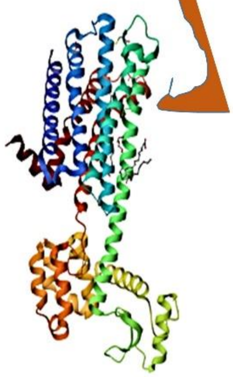

Explore in 3D: Structure | Sequence Annotations | Electron Density | Validation Report | Ligand Interaction (8NU) | Predict Membrane

6CM4

Structure of the D2 Dopamine Receptor Bound to the Atypical Antipsychotic Drug Risperidone

PDB DOI: https://doi.org/10.2210/pdb6CM4/pdb Entry: 6CM4 **supersedes:** 6C38

Classification: MEMBRANE PROTEIN
Organism(s): Homo sapiens, Tequatrovirus T4
Expression System: Spodoptera frugiperda
Mutation(s): No
Membrane Protein: Yes OPM PDBTM MemProtMD mpstruc

Deposited: 2018-03-02 **Released:** 2018-03-14
Deposition Author(s): Wang, S., Che, T., Levit, A., Shoichet, B.K., Wacker, D., Roth, B.L.
Funding Organization(s): National Institutes of Health/National Institute of Mental Health (NIH/NIMH), National Institutes of Health/National Institute of General Medical Sciences (NIH/NIGMS), National Institutes of Health/National Cancer Institute (NIH/NCI), Department of Energy (DOE, United States)

Experimental Data Snapshot

Method: X-RAY DIFFRACTION
Resolution: 2.87 Å

wwPDB Validation 3D Report Full Report

4CM6https://www.rcsb.org/structure/
