# Supplementary material for: Fabrication and appraisal of axitinib loaded PEGylated spanlastics against MCF- 7 and OV- 2774 cell lines using molecular docking methods and in-vitro study
Source: PLoS One. 2025 Jul 1;20(7):e0325055. doi: 10.1371/journal.pone.0325055 (PMC12212535; doi:10.1371/journal.pone.0325055)
Supplement: S13 Fig — (PDF) [file pone.0325055.s013.pdf]

VEGFR-Receptor

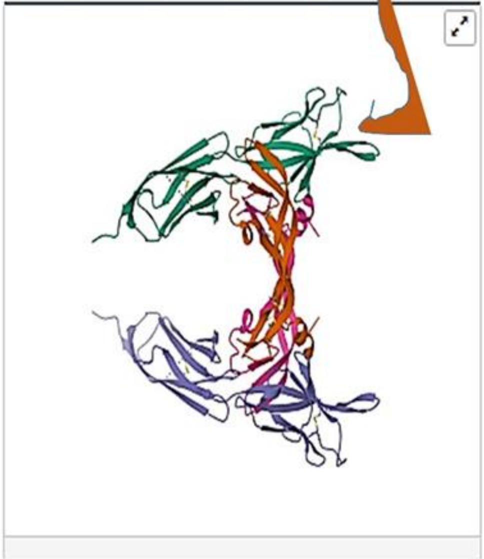

a2v3https://www.rcsb.org/structure/

3V2A

VEGFR-2/VEGF-A COMPLEX STRUCTURE

PDB DOI: https://doi.org/10.2210/pdb3V2A/pdb

Classification: HORMONE/SIGNALING PROTEIN
Organism(s): Homo sapiens
Expression System: Spodoptera frugiperda, Komagataella pastoris
Mutation(s): No

Deposited: 2011-12-12 **Released:** 2012-01-18
Deposition Author(s): Brozzo, M.S., Leppanen, V.-M., Winkler, F.K., Ballmer-Hofer, K.

Experimental Data Snapshot

Method: X-RAY DIFFRACTION
Resolution: 3.20 Å

wwPDB Validation
