# Supplementary material for: Fabrication and appraisal of axitinib loaded PEGylated spanlastics against MCF- 7 and OV- 2774 cell lines using molecular docking methods and in-vitro study
Source: PLoS One. 2025 Jul 1;20(7):e0325055. doi: 10.1371/journal.pone.0325055 (PMC12212535; doi:10.1371/journal.pone.0325055)
Supplement: S14 Fig — (PDF) [file pone.0325055.s014.pdf]

Epidermal Growth Factor receptor (EGFR)

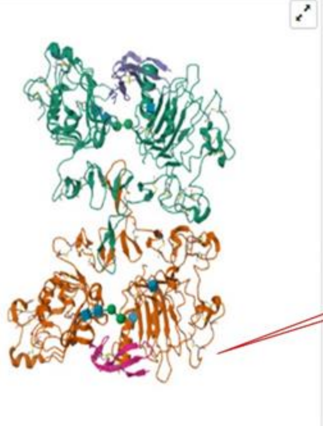

: in 3D: Structure | Sequence Annotations
Density | Validation Report |
eraction (NAG)

5WB7

Crystal structure of the epidermal growth factor receptor extracellular region in complex with epiregulin

PDB DOI: https://doi.org/10.2210/pdb5WB7/pdb

Classification: SIGNALING PROTEIN
Organism(s): Homo sapiens
Expression System: Spodoptera frugiperda, Drosophila melanogaster
Mutation(s): No

Deposited: 2017-06-28 **Released:** 2017-10-18
Deposition Author(s): Freed, D.M., Bessman, N.J., Ferguson, K.M., Lemmon, M.A.
Funding Organization(s): National Institutes of Health/National Cancer Institute (NIH/NCI), National Institutes of Health/National Institute of General Medical Sciences (NIH/NIGMS)

Experimental Data Snapshot

Method: X-RAY DIFFRACTION
Resolution: 2.94 Å

wwPDB Validation

3D Report | Full Report

Metric	Percentile Ranks	Value
Rfree		0.268

7wb5https://www.rcsb.org/structure/
