# Supplementary material for: Fabrication and appraisal of axitinib loaded PEGylated spanlastics against MCF- 7 and OV- 2774 cell lines using molecular docking methods and in-vitro study
Source: PLoS One. 2025 Jul 1;20(7):e0325055. doi: 10.1371/journal.pone.0325055 (PMC12212535; doi:10.1371/journal.pone.0325055)
Supplement: S15 Fig — (PDF) [file pone.0325055.s015.pdf]

BCL-xL-Receptor

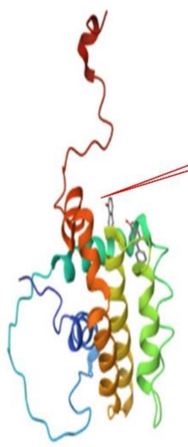

1YSG

Solution Structure of the Anti-apoptotic Protein Bcl-xL in Complex with "SAR by NMR" Ligands

PDB DOI: https://doi.org/10.2210/pdb1YSG/pdb

Classification: **APOPTOSIS**
Organism(s): Homo sapiens
Expression System: Escherichia coli BL21(DE3)
Mutation(s): No

Deposited: 2005-02-08 **Released:** 2005-06-07
Deposition Author(s): Oltersdorf, T., Elmore, S.W., Shoemaker, A.R., Armstrong, R.C., Augeri, D.J., Belli, B.A., Bruncko, M., Deckwerth, T.L., Dinges, J., Hajduk, P.J., Joseph, M.K., Kitada, S., Korsmeyer, S.J., Kunzer, A.R., Letai, A., Li, C., Mitten, M.J., Nettesheim, D.G., Ng, S., Nimmer, P.M., O'Connor, J.M., Oleksijew, A., Petros, A.M., Reed, J.C., Shen, W., Tahir, S.K., Thompson, C.B., Tomaselli, K.J., Wang, B., Wendt, M.D., Zhang, H., Fesik, S.W., Rosenberg, S.H.

ysg1https://www.rcsb.org/structure/
