# Supplementary material for: Fabrication and appraisal of axitinib loaded PEGylated spanlastics against MCF- 7 and OV- 2774 cell lines using molecular docking methods and in-vitro study
Source: PLoS One. 2025 Jul 1;20(7):e0325055. doi: 10.1371/journal.pone.0325055 (PMC12212535; doi:10.1371/journal.pone.0325055)
Supplement: S16 Fig — (PDF) [file pone.0325055.s016.pdf]

Dopamine/active site

```
Query  .....40.........50.........60.........70.........80.........90.........100.....[illegible].........120.........130...............150.........160.....
A      NYYATL LTLLIAVIVF GNVLVCMAVS REKALQTTTN YLIVSLAVAD LLVATLVMPW VVYLEVVGEW KFSR[illegible]DIF VTLDVMMCTA SALNLCAISI DRYTAVAMPTRYSSKR RVTVMISIVW VLSFT
       ....170.........180.........190.........200.........210.........220.......[illegible].1010.........1020.........1030.........1040.........1050.........
       ISCPL LFGLNNADQN ECIIANPAFV VYSSIVSFYV PFIVTLLVYI KIYIVLRRRR KRNIFEMLRID EGLRLKIYKD TEGYYTIGIG HLLTKSPSLN AAKSELDKAI GRNTNGVIT
       1060.........1070.........1080.........1090.........1100.........1110.........1120.........1130.........1140.........1150.........1160.......370..
       K DEAEKLFNQD VDAAVRGILR NAKLKPVYDS LDAVRRAALI NMVFQMGETG VAGFTNSLRM LQQKRWDEAA VNLAKSRWYN QTPNRAKRVI TTFRTGTWDA YSQQKEKK AT
       .......380.........390.........400.........410.........420.........430.........440..
       QMAAIVAG VFIICWLPFF ITHILNIHCD CNIPPVLYSA FTWLGYVNSA VNPIIYTTFN IEFRKAFLKI LH
```

Attention: The chains of the uploaded protein are broken at position **A139(PRO), A144(THR), A1161(TYR), A364(SER)**! These positions are highlighted in the sequence list with **black underline**! Please check if these positions fall in the detected pockets.

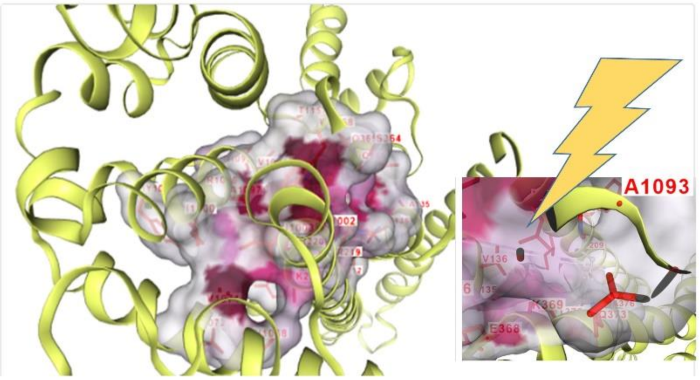

CurPocket ID	Cavity volume (Å^3)	Center (x, y, z)	Cavity size (x, y, z)
⦿C1	1620	25, 1, 23	20, 16, 19
○C2	1354	10, 4, -10	15, 14, 18
○C3	700	9, 6, 20	10, 21, 7
○C4	160	15, 17, 6	8, 6, 14
○C5	160	40, -5, 40	9, 8, 13

Download CurPockets
