# Supplementary material for: Fabrication and appraisal of axitinib loaded PEGylated spanlastics against MCF- 7 and OV- 2774 cell lines using molecular docking methods and in-vitro study
Source: PLoS One. 2025 Jul 1;20(7):e0325055. doi: 10.1371/journal.pone.0325055 (PMC12212535; doi:10.1371/journal.pone.0325055)

VEGFR/active site

```
Query   .......20.........30.........40.........50.........60.........70.........80.........90.........100.......
A       EVVKFMDV YQRSYCHPIE TLVDIFQEYP DEIEYIFKPS CVPLMRCGGC CNDEGLECVP TEESNITMQI MRIKPHQGQH IGEMSFLQHN  KCECRPK

Query   ........140.........150.........160.........170.........180.........190.........200.........210.........220.........230.........240.........250..
R       QHGVVYITE  NKNKTVVIPC  LGSISNLNVS  LCARYPEKRF  VPDGNRISWD  SKKGFTIPSY  MISYAGMVFC  EAKINDESYQ  SIMYIVVVVG  YRIYDVVLSP  SHGIELSVGE  KLVLNCTART  EL
        .......260................290.........300.........310.........320.........
        NVGIDFNW  EYPLVNRDLSEMKKFLS  TLTIDGVTRS  DQGLYTCAAS  SGLMTKKNST  FVRVHEKPF
```

Attention: The chains of the uploaded protein are broken at position **R263(PRO), R272(LEU), R277(LEU), R283(SER)**! These positions are highlighted in the sequence list with **black underline**! Please check if these positions fall in the detected pockets.

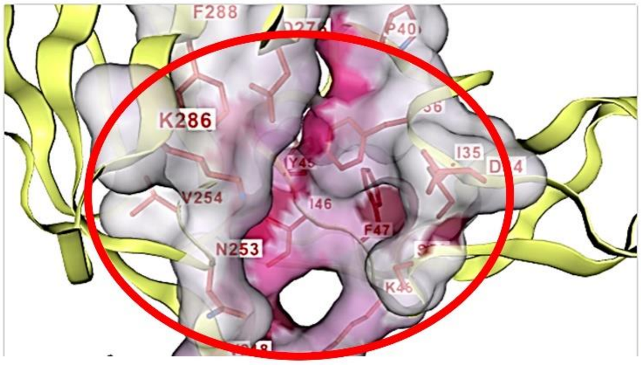

CurPocket ID	Cavity volume (Å^3)	Center (x, y, z)	Cavity size (x, y, z)
⊙C1	619	39, -10, 12	8, 21, 6
○C2	152	30, 22, 12	13, 10, 12
○C3	128	29, -3, 11	8, 7, 7
○C4	127	27, 20, 7	6, 9, 5
○C5	89	44, -11, 1	5, 7, 5

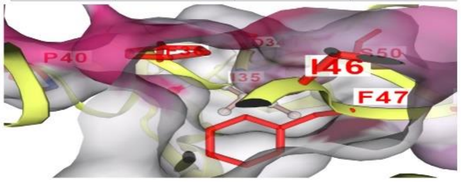

Supplement: S17 Fig — (PDF) [file pone.0325055.s017.pdf]
