# Supplementary material for: Fabrication and appraisal of axitinib loaded PEGylated spanlastics against MCF- 7 and OV- 2774 cell lines using molecular docking methods and in-vitro study
Source: PLoS One. 2025 Jul 1;20(7):e0325055. doi: 10.1371/journal.pone.0325055 (PMC12212535; doi:10.1371/journal.pone.0325055)

BCL-xL/ Active site

```
Query   .........10.........20.........30.........40.........90.........100.........110.........120.........130.........140.........150.........160......
A       MSMAMSQSNR ELVVDFLSYK LSQKGYSWSQ FSDVEENRTE APEGTESEAV KQALREAGDE FELRYRRAFS DLTSQLHITP GTAYQSFEQV VNELFRDGVN WGRIVAFFSF GGALCVESVD KEMQVL
        ...170.........180.........190.........200.........210.........220.
        VSRI AAWMATYLND HLEPWIQENG GWDTFVELYG NNAAAESRKG QERLEHHHHH H
```

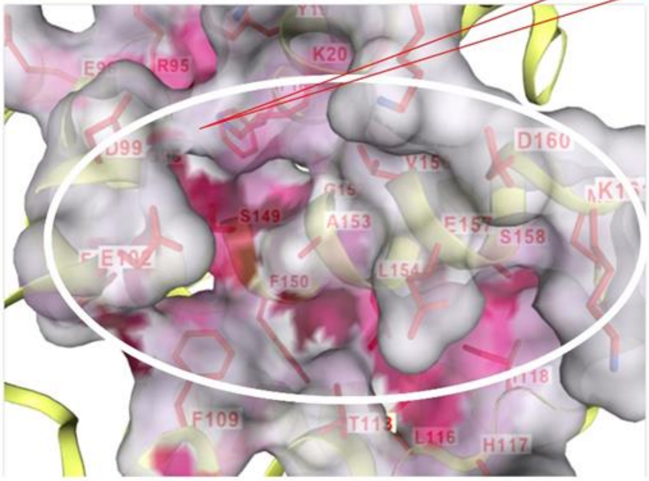

CurPocket ID	Cavity volume (Å^3)	Center (x, y, z)	Cavity size (x, y, z)
C1	2046	-6, -3, -10	23, 21, 9
C2	2045	8, 4, -4	21, 19, 15
C3	264	1, 10, 5	14, 6, 7
C4	210	6, -10, -3	8, 8, 7
C5	161	-15, 5, 3	9, 8, 9

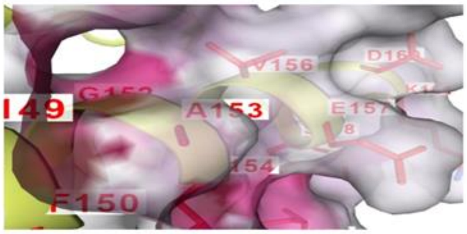

Supplement: S19 Fig — (PDF) [file pone.0325055.s019.pdf]
