# Supplementary material for: Fabrication and appraisal of axitinib loaded PEGylated spanlastics against MCF- 7 and OV- 2774 cell lines using molecular docking methods and in-vitro study
Source: PLoS One. 2025 Jul 1;20(7):e0325055. doi: 10.1371/journal.pone.0325055 (PMC12212535; doi:10.1371/journal.pone.0325055)
Supplement: S24 Fig — (PDF) [file pone.0325055.s024.pdf]

mode	affinity (kcal/mol)	dist from best mode rmsd l.b.	rmsd u.b.
1	-8.0	0.000	0.000
2	-7.7	2.254	2.852
3	-7.7	2.416	3.326
4	-7.5	17.080	19.447
5	-7.4	17.097	19.434
6	-7.4	2.269	3.043
7	-7.3	17.923	20.040
8	-7.2	18.517	20.948
9	-7.1	2.525	3.347

Axitinib & dopamine

mode	affinity (kcal/mol)	dist from best mode rmsd l.b.	rmsd u.b.
1	-2.9	0.000	0.000
2	-2.7	7.178	7.391
3	-2.7	1.384	2.146
4	-2.7	7.232	7.696
5	-2.7	1.827	2.036
6	-2.5	1.861	2.096
7	-2.5	7.314	7.861
8	-2.5	11.183	11.546
9	-2.4	23.906	24.306

PEG & Dopamine

mode	affinity (kcal/mol)	dist from best mode rmsd l.b.	rmsd u.b.
1	-7.7	0.000	0.000
2	-7.5	8.770	13.184
3	-7.2	1.820	2.170
4	-7.2	2.010	3.021
5	-7.2	7.675	12.998
6	-7.0	5.238	11.966
7	-6.9	11.873	15.632
8	-6.8	4.387	10.214
9	-6.8	2.024	2.275

Axitinib & VEGFR

mode	affinity (kcal/mol)	dist from best mode rmsd l.b.	rmsd u.b.
1	-3.1	0.000	0.000
2	-2.9	9.204	9.687
3	-2.9	9.145	9.663
4	-2.9	0.461	2.048
5	-2.8	1.491	2.027
6	-2.4	14.614	15.148
7	-2.4	3.639	3.935
8	-2.4	14.940	15.404
9	-2.3	20.579	21.210

PEG & VEGFR
