# Supplementary material for: Fabrication and appraisal of axitinib loaded PEGylated spanlastics against MCF- 7 and OV- 2774 cell lines using molecular docking methods and in-vitro study
Source: PLoS One. 2025 Jul 1;20(7):e0325055. doi: 10.1371/journal.pone.0325055 (PMC12212535; doi:10.1371/journal.pone.0325055)
Supplement: S25 Fig — (PDF) [file pone.0325055.s025.pdf]

mode	affinity (kcal/mol)	dist from best mode rmsd l.b.	rmsd u.b.
1	-2.8	0.000	0.000
2	-2.7	2.982	4.253
3	-2.6	2.300	3.290
4	-2.6	3.113	9.539
5	-2.5	2.696	4.007
6	-2.5	3.461	6.223
7	-2.5	2.864	4.186
8	-2.4	1.732	2.427
9	-2.3	2.995	9.325

Axitinib & BCL-xL

mode	affinity (kcal/mol)	dist from best mode rmsd l.b.	rmsd u.b.
1	-1.5	0.000	0.000
2	-1.5	12.076	12.720
3	-1.4	0.705	2.218
4	-1.4	2.522	3.438
5	-1.3	2.683	3.232
6	-1.3	1.893	2.584
7	-1.3	2.990	3.214
8	-1.3	1.829	2.297
9	-1.1	2.196	2.203

PEG & BCL-xL

mode	affinity (kcal/mol)	dist from best mode rmsd l.b.	rmsd u.b.
1	-8.3	0.000	0.000
2	-8.0	2.008	2.375
3	-7.9	4.729	9.850
4	-7.9	7.528	10.756
5	-7.9	7.936	9.667
6	-7.8	7.810	9.745
7	-7.6	7.840	12.830
8	-7.6	3.564	8.890
9	-7.5	8.074	10.284

Axitinib & EGFR

mode	affinity (kcal/mol)	dist from best mode rmsd l.b.	rmsd u.b.
1	-3.3	0.000	0.000
2	-3.1	1.930	2.478
3	-3.1	1.925	2.452
4	-3.1	1.978	2.660
5	-2.7	24.518	24.829
6	-2.7	23.656	23.952
7	-2.7	18.165	18.389
8	-2.7	14.847	15.391
9	-2.6	14.810	15.426

PEG & EGFR
