# Supplementary material for: Fabrication and appraisal of axitinib loaded PEGylated spanlastics against MCF- 7 and OV- 2774 cell lines using molecular docking methods and in-vitro study
Source: PLoS One. 2025 Jul 1;20(7):e0325055. doi: 10.1371/journal.pone.0325055 (PMC12212535; doi:10.1371/journal.pone.0325055)
Supplement: S27 Fig — (PDF) [file pone.0325055.s027.pdf]

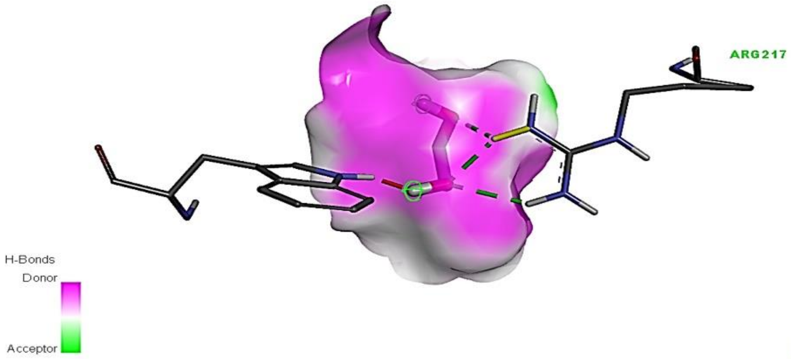

	Name	Visible	Color	Parent	Distance	Category	Types	From	From Chemistry	To	To Chemistry	Angle DHA	Angle HAY
1	A:ARG21...	☑Yes		Ligand No...	2.41364	Hydrogen Bo...	Conventi...	A:ARG2...	H-Donor	:UNL...	H-Acceptor	142.236	104.537
2	A:ARG21...	☑Yes		Ligand No...	2.24736	Hydrogen Bo...	Conventi...	A:ARG2...	H-Donor	:UNL...	H-Acceptor	114.384	109.702
3	A:ARG21...	☑Yes		Ligand No...	2.1022	Hydrogen Bo...	Conventi...	A:ARG2...	H-Donor	:UNL...	H-Acceptor	148.697	112.085

3D interaction & shared Amino acids of Dopamine &PEG

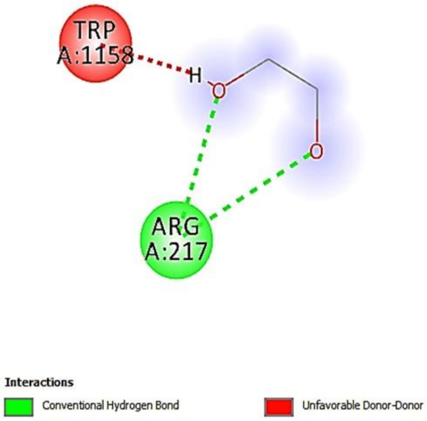

2D interaction –Dopamine &PEG
