# Supplementary material for: Fabrication and appraisal of axitinib loaded PEGylated spanlastics against MCF- 7 and OV- 2774 cell lines using molecular docking methods and in-vitro study
Source: PLoS One. 2025 Jul 1;20(7):e0325055. doi: 10.1371/journal.pone.0325055 (PMC12212535; doi:10.1371/journal.pone.0325055)
Supplement: S28 Fig — (PDF) [file pone.0325055.s028.pdf]

VEGFR &Axitinib

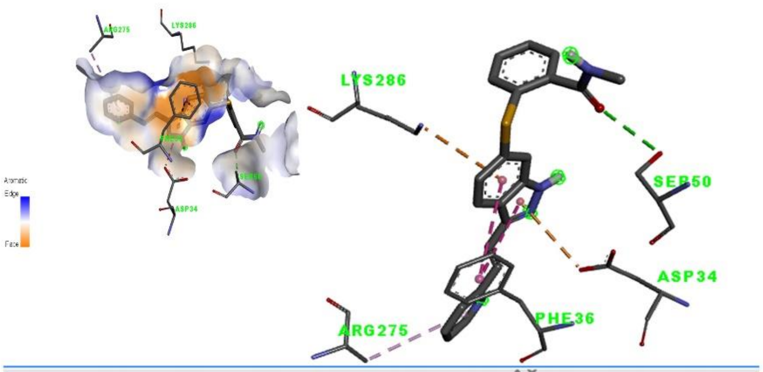

Name	Visible	Color	Parent	Distance	Category	Types	From	From Chemistry	To	To Chemistry
A:SER50:...	Yes		Ligand No...	3.1389	Hydrogen Bo...	Conventi...	A:SER50...	H-Donor	:UNL...	H-Acceptor
R:LYS286...	Yes		Ligand No...	4.07793	Hydrogen Bo...	Pi-Cation...	R:LYS28...	Positive;H-Donor	:UNL1	Pi-Orbitals;Pi-Orb...
A:ASP34:...	Yes		Ligand No...	4.02913	Electrostatic	Pi-Anion	A:ASP34...	Negative	:UNL1	Pi-Orbitals
A:PHE36 ...	Yes		Ligand No...	4.71494	Hydrophobic	Pi-Pi Stac...	A:PHE36	Pi-Orbitals	:UNL1	Pi-Orbitals
:UNL1 - A...	Yes		Ligand No...	5.35829	Hydrophobic	Pi-Pi Stac...	:UNL1	Pi-Orbitals	A:PH...	Pi-Orbitals
:UNL1 - R...	Yes		Ligand No...	4.87277	Hydrophobic	Pi-Alkyl	:UNL1	Pi-Orbitals	R:AR...	Alkyl

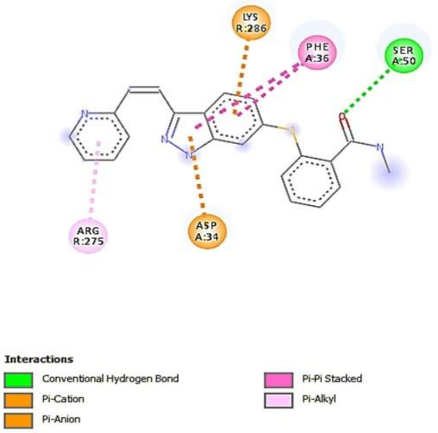

2D interaction -VEGFR & Axitinib

3D interaction & shared Amino acids of VEGFR & Axitinib
