# Supplementary material for: Fabrication and appraisal of axitinib loaded PEGylated spanlastics against MCF- 7 and OV- 2774 cell lines using molecular docking methods and in-vitro study
Source: PLoS One. 2025 Jul 1;20(7):e0325055. doi: 10.1371/journal.pone.0325055 (PMC12212535; doi:10.1371/journal.pone.0325055)
Supplement: S1 Appendix — (DOCX) [file pone.0325055.s034.docx]

**
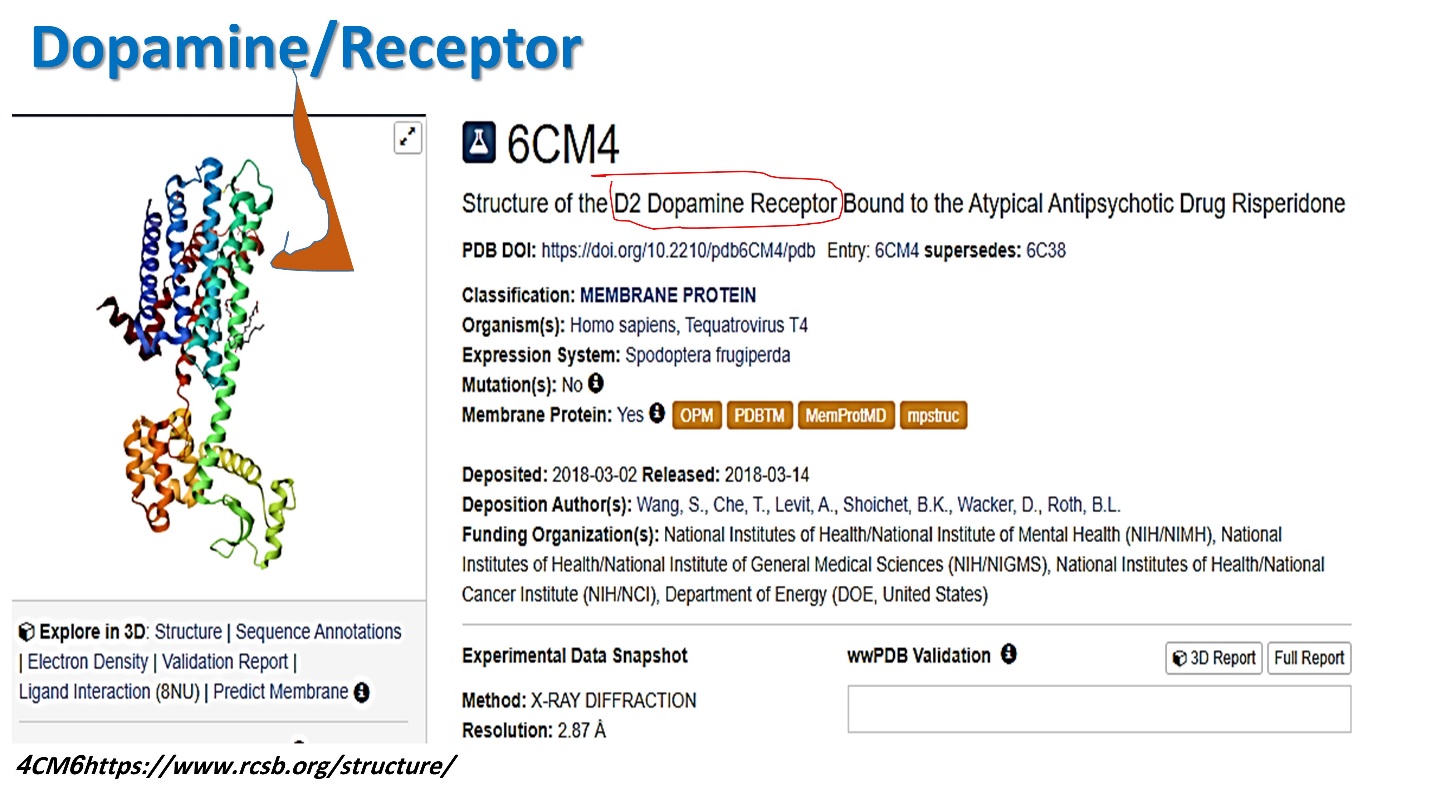
**

Figure S 1: Dopamine Receptor.pdb.id & chemical structure (6CM4) Structure, ID code and origin or source of protein with the shared amino acids.


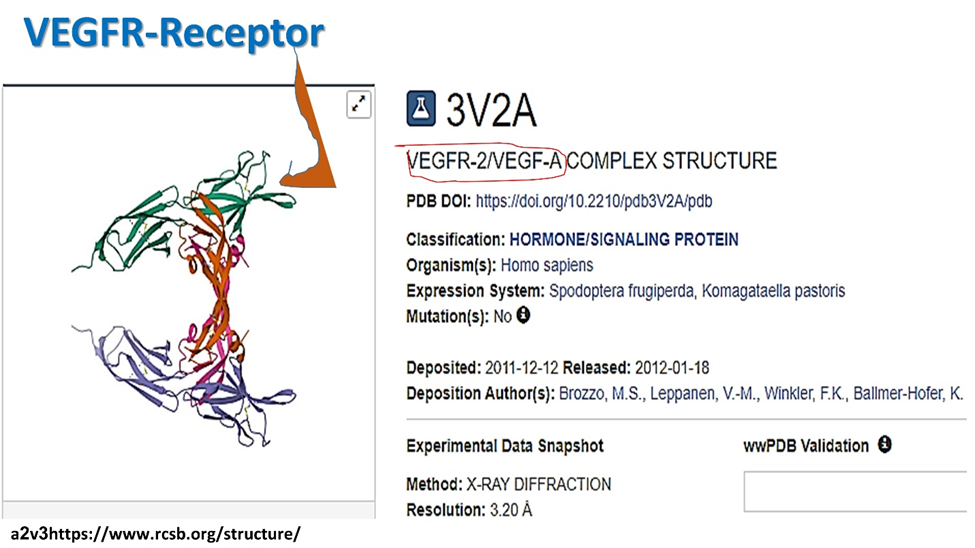


Figure S 2: VEGF Receptor.pdb.id & chemical structure (3V2A) Structure, ID code and origin or source of protein with the shared amino acids.


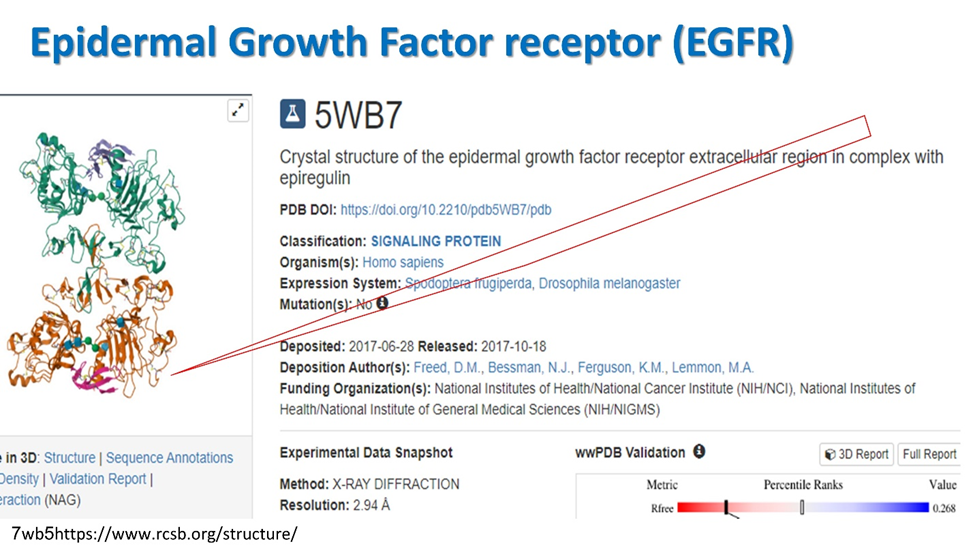


Figure S 3: EGFR Receptor.pdb.id & chemical structure (5WB7) Structure, ID code and origin or source of protein with the shared amino acids.


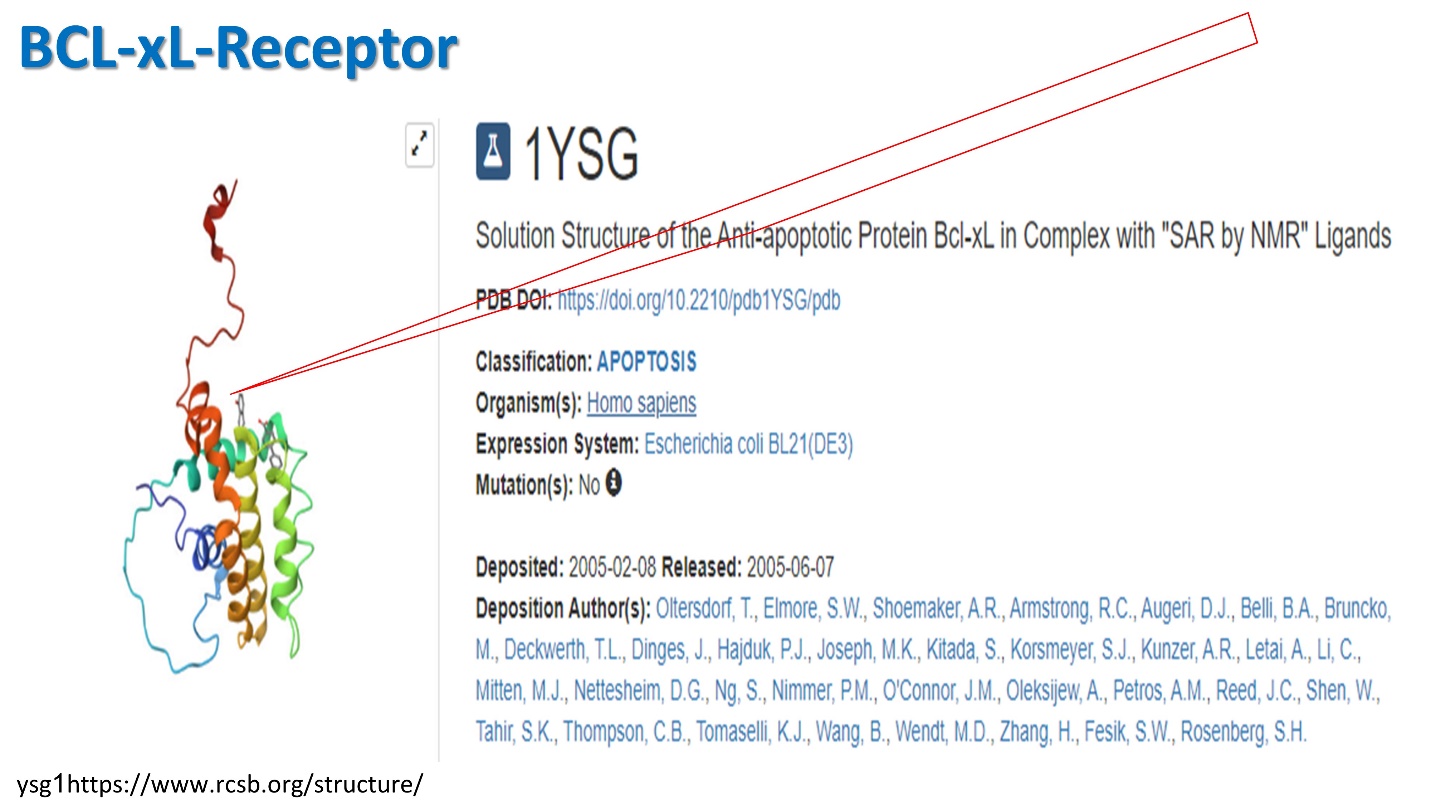


Figure S 4: BCL-xL Receptor.pdb.id & chemical structure (1YSG) Structure, ID code and origin or source of protein with the shared amino acids.


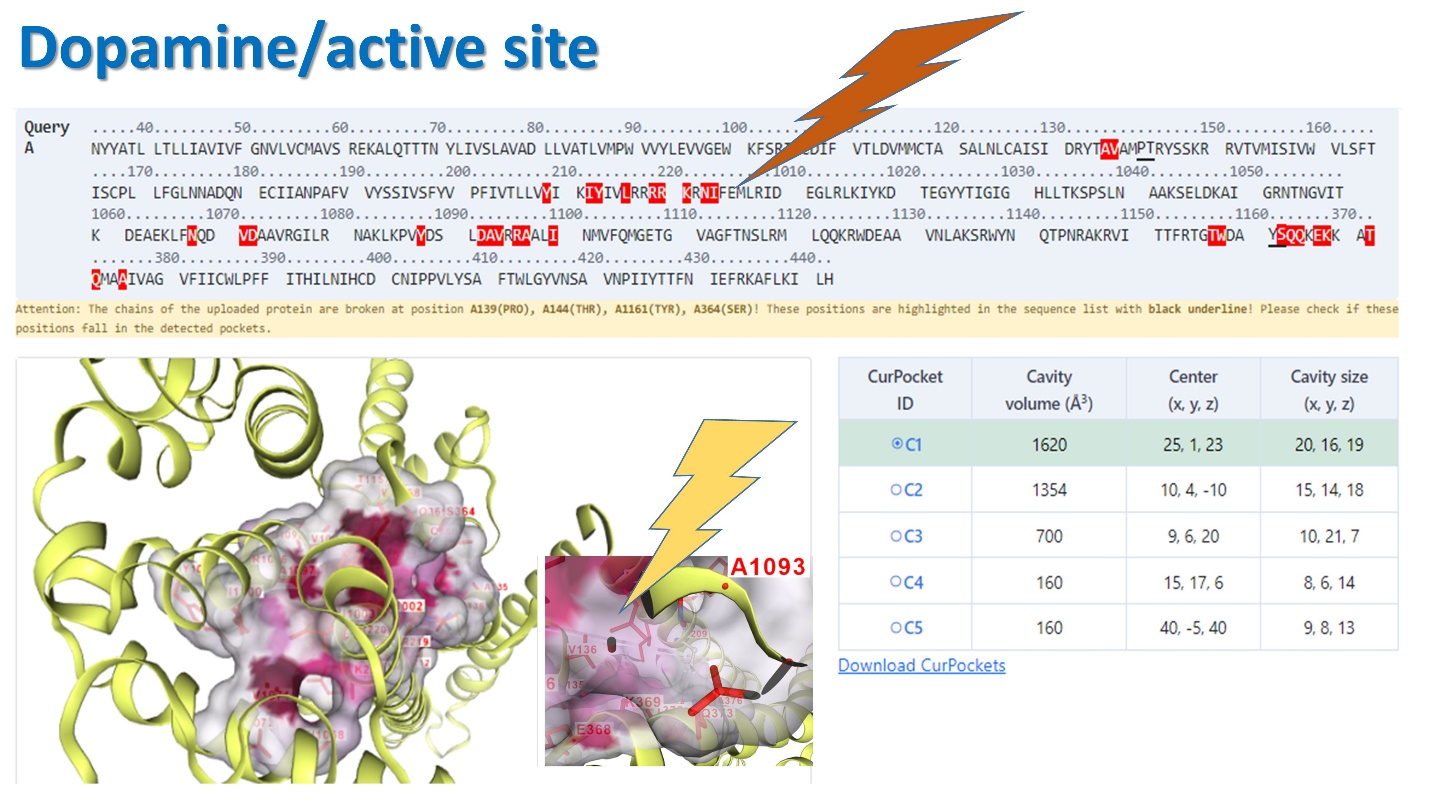


Figure S 5: Pocket, target of Dopamine protein and its shared amino acids in the active site of binding with axitinib.


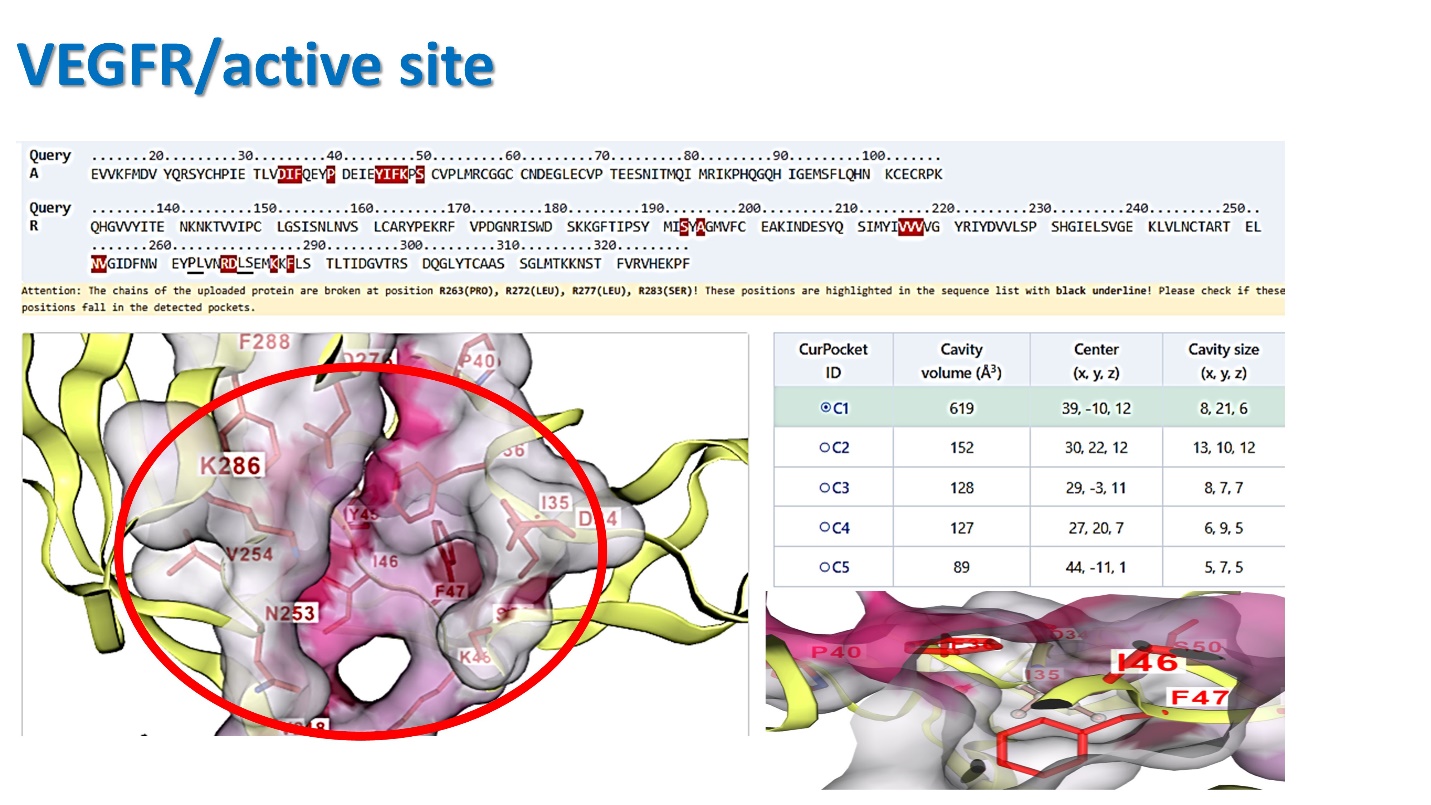


Figure S 6: Pocket, target of VEGFR protein and its shared amino acids in the active site of binding with axitinib.


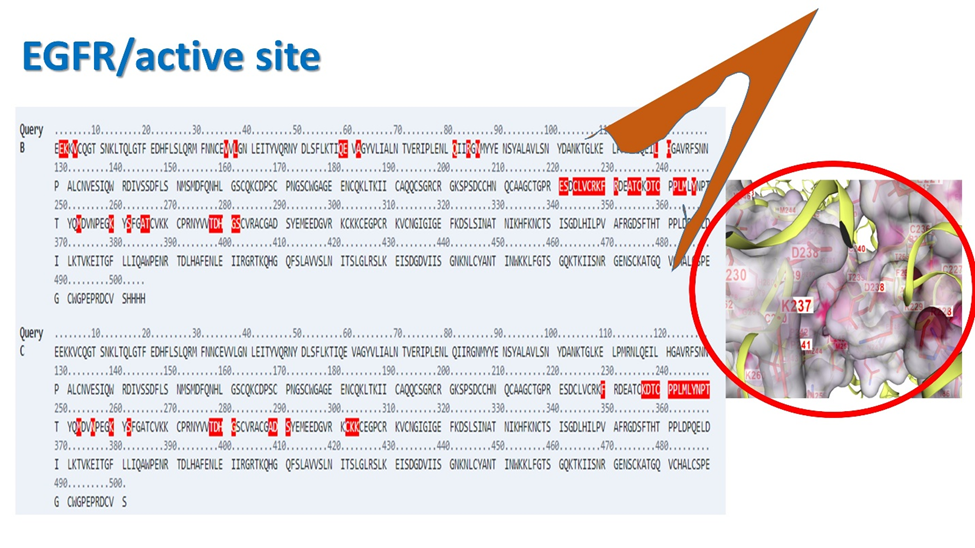


Figure S 7: Pocket, target of EGFR protein and its shared amino acids in the active site of binding with axitinib.


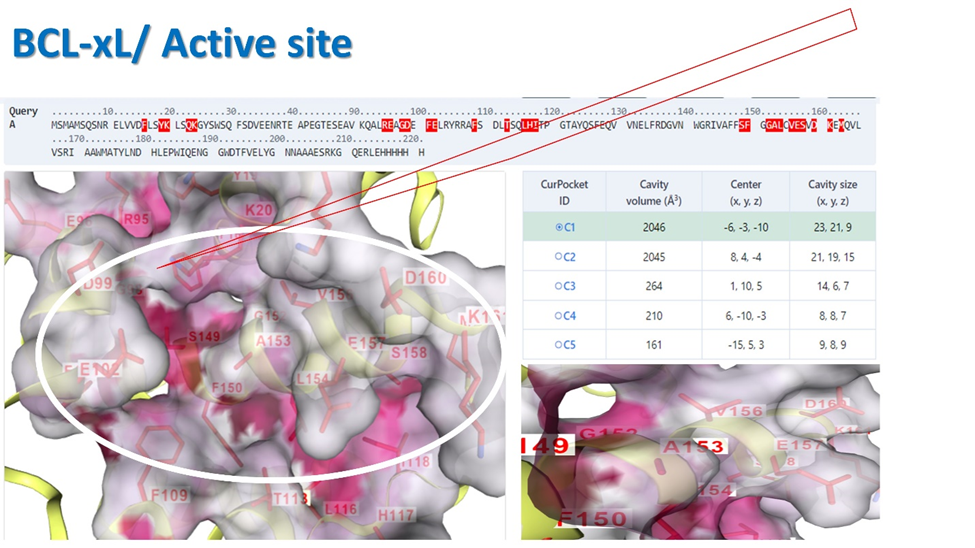


Figure S 8: Pocket, target of BCL-xL protein and its shared amino acids in the active site of binding with axitinib.
