# Supplementary material for: Fabrication and appraisal of axitinib loaded PEGylated spanlastics against MCF- 7 and OV- 2774 cell lines using molecular docking methods and in-vitro study
Source: PLoS One. 2025 Jul 1;20(7):e0325055. doi: 10.1371/journal.pone.0325055 (PMC12212535; doi:10.1371/journal.pone.0325055)
Supplement: S1 File — (PDF) [file pone.0325055.s035.pdf]

	Plain	Axitinib suspension	Axitinib spanlastic	Axitinib-PEG-spanlastic
MCF-7	13.74	32.6	36.33	70.76
MCF-7	15.44	31.5	35.23	67.5
Average	14.59	32.05	35.78	69.13

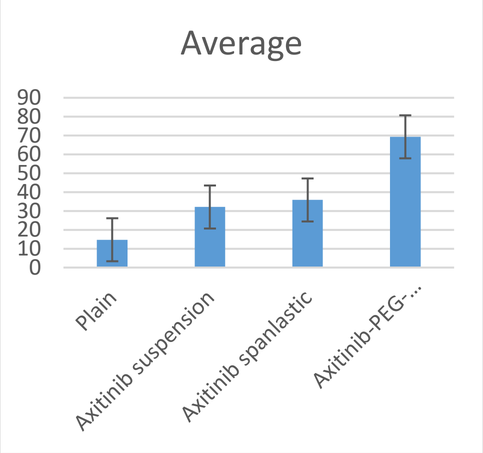

Anova: Single Factor

SUMMARY

Groups	*Count*	*Sum*	*Average*	*Variance*
Plain	2	29.48	14.74	2
Axitinib suspension	2	64.2	32.1	0.5
Axitinib spanlastic	2	71.66	35.83	0.5
Axitinib-PEG-spanlastic	2	138.62	69.31	4.205

ANOVA

Source of Variation	*SS*	*df*	*MS*	*F*	*P-value*	*F crit*
Between Groups	3121.725	3	1040.575	577.696	9.9345E-06	6.591382
Within Groups	7.205	4	1.80125			
Total	3128.93	7				

Anova: Single Factor

SUMMARY

Groups	*Count*	*Sum*	*Average*	*Variance*
Axitinib suspension	2	64.2	32.1	0.5
Axitinib spanlastic	2	71.66	35.83	0.5
Axitinib-PEG-spanlastic	2	138.62	69.31	4.205

ANOVA

Source of Variation	SS	df	MS	F	P-value	F crit
Between Groups	1679.605	2	839.8025	484.036	0.00017171	9.552094
Within Groups	5.205	3	1.735			
Total	1684.81	5				

Anova: Single Factor

SUMMARY

Groups	Count	Sum	Average	Variance
Axitinib spanlastic	2	71.66	35.83	0.5
Axitinib-PEG-spanlastic	2	138.62	69.31	4.205

ANOVA

Source of Variation	SS	df	MS	F	P-value	F crit
Between Groups	1120.91	1	1120.91	476.4763	0.00209216	18.51282
Within Groups	4.705	2	2.3525			
Total	1125.615	3				
