# Supplementary material for: Fabrication and appraisal of axitinib loaded PEGylated spanlastics against MCF- 7 and OV- 2774 cell lines using molecular docking methods and in-vitro study
Source: PLoS One. 2025 Jul 1;20(7):e0325055. doi: 10.1371/journal.pone.0325055 (PMC12212535; doi:10.1371/journal.pone.0325055)
Supplement: S2 File — (PDF) [file pone.0325055.s036.pdf]

	Plain	Axitinib suspension	Axitinib spanlastic	Axitinib-PEG-spanlastic
OV-2775	12.48	24.44	35.22	43.55
OV-2776	16.49	22.11	33.15	41.61
Average	14.485	23.275	34.185	42.58

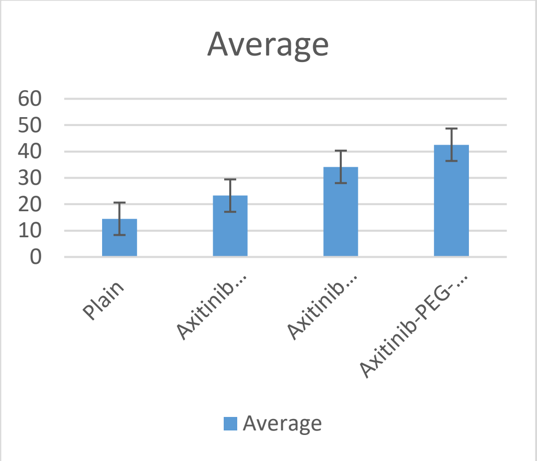

Anova: Single Factor

SUMMARY

Groups	*Count*	*Sum*	*Average*	*Variance*
Plain	2	28.96	14.48	8
Axitinib suspension	2	46.65	23.325	2.48645
Axitinib spanlastic	2	68.36	34.18	2.1632
Axitinib-PEG-spanlastic	2	85.06	42.53	2.0808

ANOVA

Source of Variation	*SS*	*df*	*MS*	*F*	*P-value*	*F crit*
Between Groups	904.756	3	301.5853	81.8944	0.000479	6.591382
Within Groups	14.73045	4	3.682613			
Total	919.4865	7				

Anova: Single Factor

SUMMARY

Groups	*Count*	*Sum*	*Average*	*Variance*
Axitinib suspension	2	46.65	23.325	2.48645
Axitinib spanlastic	2	68.36	34.18	2.1632
Axitinib-PEG-spanlastic	2	85.06	42.53	2.0808

ANOVA

Source of Variation	SS	df	MS	F	P-value	F crit
Between Groups	370.9237	2	185.4619	82.66692	0.002379	9.552094
Within Groups	6.73045	3	2.243483			
Total	377.6542	5				

Anova: Single Factor

SUMMARY

Groups	Count	Sum	Average	Variance
Axitinib spanlastic	2	68.36	34.18	2.1632
Axitinib-PEG-spanlastic	2	85.06	42.53	2.0808

ANOVA

Source of Variation	SS	df	MS	F	P-value	F crit
Between Groups	69.7225	1	69.7225	32.85697	0.029112	18.51282
Within Groups	4.244	2	2.122			
Total	73.9665	3				
